# Enablers of and barriers to implementation of school health services: A scoping review

**DOI:** 10.1371/journal.pone.0361415

**Published:** 2026-11-13

**Authors:** Diane Davis, Beth Jameson, Anita Moyes, Kathleen Butler, Nancy Ann Innella, Loree S. LaChance, Karen S. Schwind, Titus Divala, Valentina Baltag, Erin D. Maughan

**Affiliations:** 1 School of Nursing, Georgetown University, Washington, District of Columbia, United States of America; 2 Morristown Medical Center, Morristown, New Jersey, United States; 3 School of Nursing and Midwifery, Edith Cowan University, Joondalup, Western Australia, Australia; 4 Fenwick Library, George Mason University, Fairfax, Virginia, United States; 5 College of Nursing, Seton Hall University, South Orange, New Jersey, United States of America; 6 LaChance School Health Strategies, LLC, Driftwood, Texas, United States; 7 Health Services, New Braunfels Independent School District, New Braunfels, Texas, United States; 8 Adolescent and Young Adult Health Unit, Department of Maternal, Newborn, Child, Adolescent Health and Ageing, World Health Organization, Geneva, Switzerland; 9 Adolescent and Young Adult Health Unit, Department of Maternal, Newborn, Child, Adolescent Health and Ageing, World Health Organization, Geneva, Switzerland; 10 George Mason University & Center for School Health Innovation & Quality, Fairfax, Virginia, United States of America; Addis Ababa University, ETHIOPIA

## Abstract

Schools have an extraordinary potential to provide intensive, long-term, and large-scale health programs to children and adolescents. Global and regional observations of School Health Services (SHS) indicate that many SHS programs lack an evidence-base, are underfunded, and/or are delivered with limited reach and scope. This scoping review was undertaken to identify enablers of and barriers to implementation of SHS. Databases and grey literature were searched between 2000–2025 (August) to include enablers and barriers to the implementation of SHS delivered by a health worker. Studies were obtained from all World Bank regions. Of 13,278 records obtained via databases, 45 were retained for final review. Of 215 records obtained via other methods, 14 were retained. Data extraction was organized into 12 categories, based on the Health Promoting Schools framework with additional thematic domains added to further explore gaps and needs for the effective implementation of SHS. Evidence was assessed using the Melnyk & Fineout-Overholt Hierarchy of Evidence Grading Framework. Findings support several key enablers. Intersectoral collaboration grounded in a shared vision that integrated health and education was critical. Strong policy frameworks aligning policy at all levels while accounting for diverse cultural, contextual and resource environments were key to providing necessary structure. Stakeholder engagement that included health professionals, school administration, educators and school staff, parents, students and the wider community contributed to meaningful health programs that addressed local needs. Robust funding mechanisms were needed to support sufficient workforce and adequate physical infrastructure. Workforce capacity building was necessary to support new roles and address local challenges in real time. Intentional evaluation of successful SHS programs in all countries is needed for future publications. The results of this review provide policy makers, practitioners and other stakeholders with a rich description of strategies that have supported successful implementation of SHS globally.

## Introduction

Schools have an extraordinary potential to provide intensive, long-term, and large-scale health programs to children and adolescents. Globally, most children and adolescents are enrolled in school, and an increasing proportion of students continue enrollment from primary to secondary school. A system of school health services (SHS) may be the only institutional means to meet the health needs of most school-age children and adolescents on a daily basis. Additionally, SHS may also be a highly effective means to support schools in the task of preparing children and young people to be educated, productive citizens.

The World Health Organization (WHO) defines SHS as “services provided by a health worker to students enrolled in primary or secondary education, either within school premises or in a health service situated outside the school” [1]. A health worker is a licensed health professional such as a nurse, medical doctor, dentist, speech-language therapist, or psychologist.

In 2021, the WHO, in collaboration with the United Nations Educational, Scientific and Cultural Organization (UNESCO) and other partners, published a package of guidance to support SHS, including global standards for health promoting schools (HPS) [1–4]. However, global and regional observations of SHS indicate that while most countries have some form of SHS, many programs lack an evidence-base, are underfunded, and/or are delivered with limited reach and scope [1]. This scoping review was undertaken to identify enablers of and barriers to implementation of SHS.

### Research question

What are the enablers of and barriers to the implementation of school health services delivered by health workers in primary and secondary schools globally?

## Methods

This scoping review was conducted according to the preferred reporting items for systematic reviews and meta-analysis (PRISMA) [5] and meets criteria outlined in the PRISMA extension for scoping reviews. See S4 File. The research question was provided by the WHO (VB & TD), who also reviewed the search protocol before the process began. A protocol was developed *a priori* that outlined context, objectives, operational definitions, search strategy, inclusion and exclusion criteria and quality appraisal methods. This document was followed throughout the review process.

### Search strategy

Databases and grey literature sources were systematically searched using a search strategy created by an academic medical librarian and peer reviewed by a librarian colleague. The search strategy is presented in S1 File. Date limits were 2000–2025 (August) to correspond to the time period following the announcement of the United Nations Millennium Development Goals in 2000. The original search date limits were 2000–2023 (October) and an additional search to update for recency once the original analysis was complete were 2023–2025 (August). No limits on language were imposed. In addition, the WHO shared a database of national and subnational school health documents from past searches that was reviewed.

### Eligibility criteria

To be included in this review, articles needed to include barriers, challenges, facilitators and enablers associated with implementation of SHS, and describe services delivered in a primary and/or secondary school during (or predominantly during) school hours. The articles could report on experimental or non-experimental studies of any design that examined factors (enablers or barriers) qualitatively and/or quantitatively related to the aspects of SHS policy, practice, or program implementation at all levels: schools, state, province, county, country, national, or international. Study participants could be any end-user or stakeholder who was involved in the implementation of SHS, including teachers, principals, service managers or other relevant staff, parents, guardians, children, community partners, administrators or officials of other government or nongovernment agencies that regulate, encourage, or enforce health in schools. Authors of included articles were not required to be health workers, however the programs described needed to align with the WHO definition of SHS, that is, services delivered by a health worker. Articles could be in any language. Exclusion criteria were childcare, daycare, and preschool and any level of schooling that was not primary or secondary education. Articles that did not address the WHO definition of SHS (i.e., health services/education delivered by other than a health care worker or delivered to students other than those enrolled in primary and secondary education), and articles that were commentaries about implementation were also excluded.

### Databases and sources

Databases searched were PubMed, Cumulative Index to Nursing and Allied Health Literature (CINAHL), Global Health, Educational Resources Information Center (ERIC), Cochrane Central Register of Controlled Trials (CENTRAL), and Web of Science. The search statements for CINAHL and PubMed used both keywords and Medical Subject Headings (MeSH), and the other database searches used keywords only. In addition, Latin American and Caribbean Literature on Health Sciences (LILACS), Global Index Medicus and Google were searched to retrieve non-English language articles not indexed in other databases. The librarian used a three-pronged approach (adapted from Godin et al., 2015) [6] to find grey literature on SHS implementation: grey literature databases, general and customized Google searches, and targeted websites. Grey literature databases searched were Policy Commons, Bielefeld Academic Search Engine (BASE), Proquest Theses & Dissertations, OAIster, WHO Library Database, WHO website, Global Index Medicus and World Bank and Google.

### Search methods

Searches of the peer-reviewed literature and grey literature were conducted according to PRISMA guidelines [5]. See Fig 1 and Fig 2. The original searches for peer-reviewed literature were executed in each database and the resulting citations (11,915) were exported first to Zotero to remove duplicates, then 5838 citations were imported into Rayyan. Two systematic reviews were identified in the results, and the librarian used citation mining to compare the bibliographies to the articles retrieved in the searches. All but five articles were duplicates, and these five articles were added to the total and reviewed according to the screening procedures. To identify grey literature, individual Google searches combined keywords “school health services” or “school-based health” with the names of countries listed in WHO directories, or “ministry of health”, or keywords reflecting implementation. Although a wide range of concepts were searched and no limits were put on language, all search terms were in English, hampering the ability to capture non-English language publications. Results of the grey literature searches were entered into an Excel spreadsheet, with separate sheets for Country Search (Google), General Google Search, Webinars, WHO Documents, and BASE Clearinghouse. Each entry included the title and Uniform Resource Locator (URL) for the originating site.

**Fig 1:**
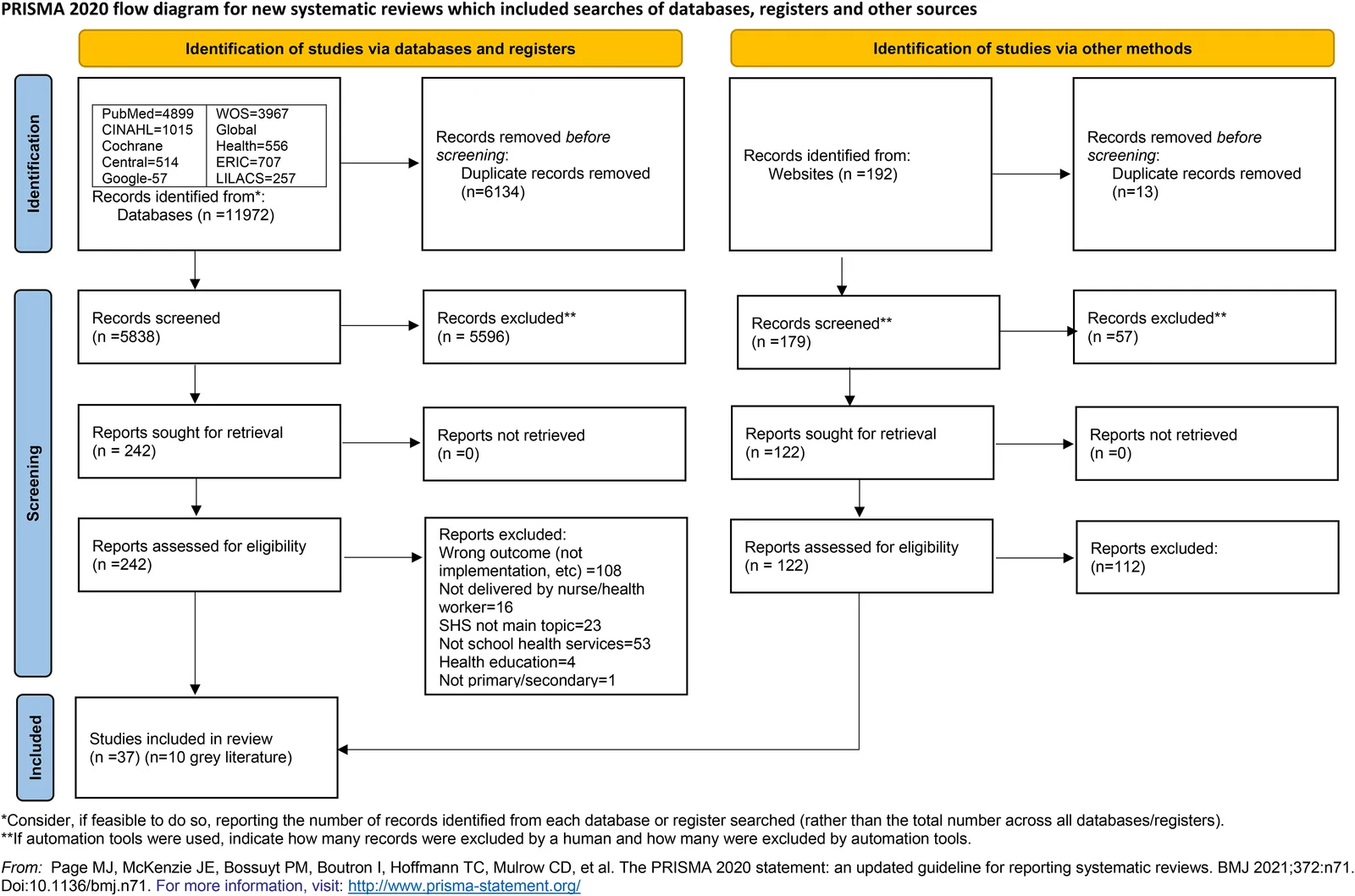
PRISMA flow diagram.

**Fig 2:**
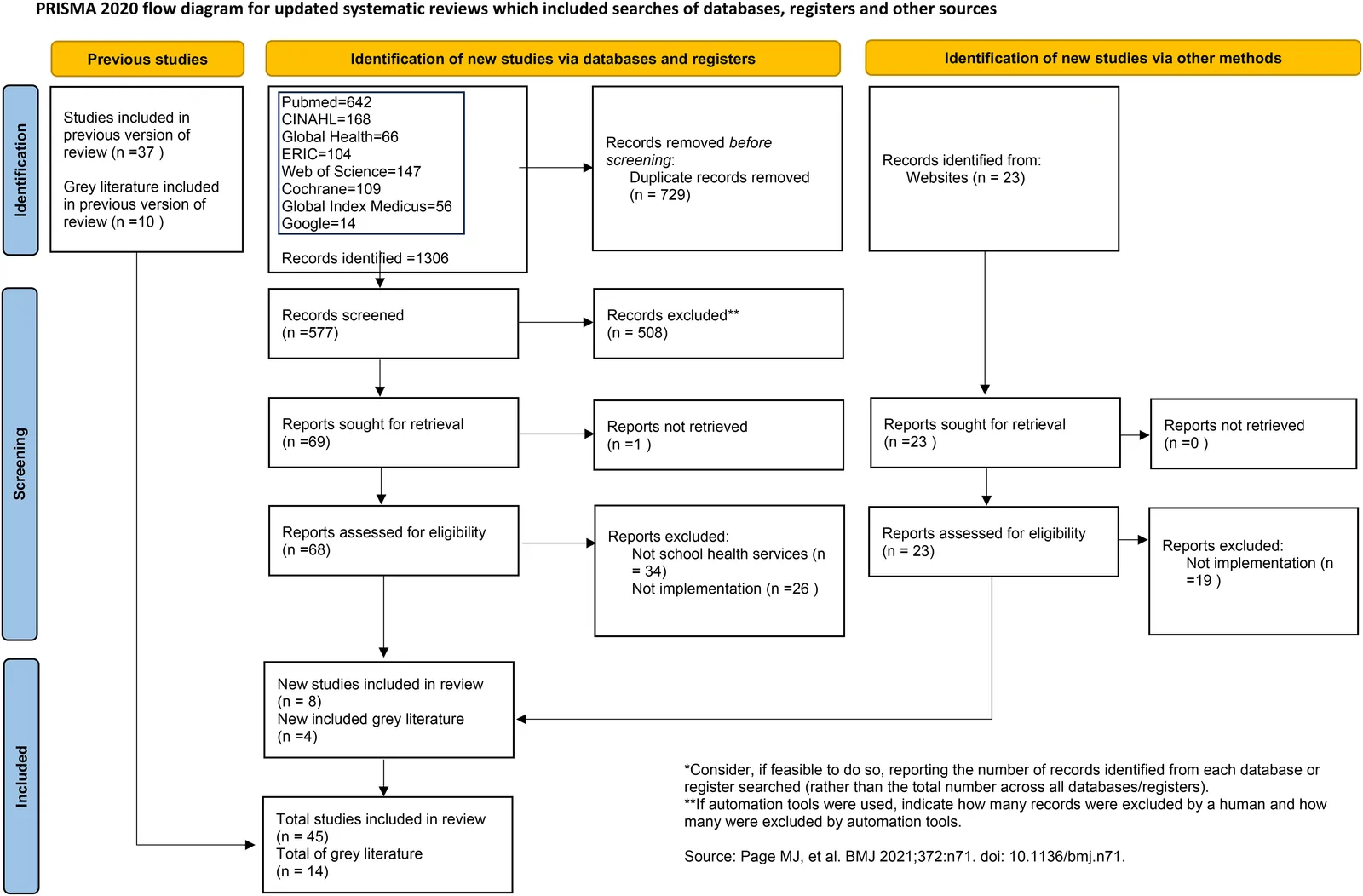
Updated PRISMA flow diagram.

### Assessment for eligibility and selection

#### Peer-reviewed publications.

In Rayyan, three pairs of reviewers screened, with blinding on, initial sets of articles for calibration and to standardize language for exclusion labels. The 5838 journal articles were then divided into three sets of 1900 + citations and each set was assigned to a team of two reviewers (BJ & AM, DD & LL, KS & NI). The three teams of two reviewers each evaluated titles and abstracts with blinding on. Once both reviewers completed their reviews, the blind was lifted so they could compare decisions. Conflicts were resolved with team discussions. If consensus could not be reached, a third reviewer (EM) was asked to independently review the title and abstract and make the final determination of eligibility. Each team met to discuss conflicts at points during the review process, and all reviewers met together to bring questions and problems to the entire group. Teams used eligibility criteria when evaluating each title and abstract in the Rayyan set and some entries had more than one reason for exclusion. Major reasons for exclusion during title and abstract screening were: Not SHS 36%; Not SHS implementation 29%; Not delivered by nurse or health worker 16%; Health education not health services 6%; Not primary or secondary education, 3.3%; Protocol 1.2%. After initial screening, a new Rayyan folder was created with the final articles for full-text review for each team. The librarian uploaded and attached full-text to each citation in Rayyan. Reviewers made final eligibility determinations based on a full document review. For non-English language articles, an English abstract was uploaded to Rayyan. Google Translate was used for full document review of non-English language articles. A final Rayyan folder was created with the remaining documents selected for inclusion. The final documents were reviewed by two authors (BJ & EM) to ensure all met criteria. Of the 242 articles selected for full text review, reasons for exclusion were: Wrong outcome (not implementation, etc.) = 108; Not delivered by a health worker = 16; SHS not main topic = 23; Not school health services = 53; Health education = 4; Not primary or secondary education = 1. Three of the retained articles were not peer reviewed literature; two were published dissertations and one was a published thesis. Thirty-seven articles were included in the review.

An update was conducted to include literature published after the initial search on October 2023, to August 2025. Using the same process, 1306 articles were identified, 68 articles were assessed for eligibility and 8 included for the final review. Reasons for exclusion were: Not SHS (n = 34) and Not SHS implementation (n = 26).

#### Grey literature.

Determination of eligibility of the grey literature entries was made by two authors (BJ & EM) using the same criteria. Ten grey literature documents were included in the original review. An update (August 2025) was conducted using the same process and criteria and four documents were included in the final review.

#### Analysis framework and data extraction tables.

Data extraction from the included peer-reviewed and grey literature was conducted independently. The included articles were assigned to specific authors (BJ, AM, DD, LL, KS & NI) to extract the data and later reviewed by at least one other author (BJ, DD & EM). Data extraction sheets were prepared using PRISMA guidelines [5]. The following information was extracted from the peer-reviewed literature: author(s), year of publication, country, framework, research aims, school level/type, research methods, number and type of respondent. The following information was extracted from the grey literature: authors, country, title, year, type of document, geographic coverage, document summary.

To initially organize data pertaining to the enablers and barriers, the extraction sheets included 12 categories, established *a priori*. Nine of the category items were identified using the framework initially described by Samdal and Rowling [7] to capture the theoretical and empirical base for Health Promoting Schools (HPS). Three additional items, identified in consultation with WHO researchers (VB & TD), were added to capture thematic domains to further explore gaps and needs for the effective implementation of SHS. The final data extraction table, provided by the WHO (VB & TD) from the outset of the study, included the nine implementation constructs aligned with the HPS framework: 1) preparing and planning for SHS, 2) policy and institutional anchoring of SHS, 3) learning and management practices, 4) school physical environment, 5) school social environment (relational and organizational context), 6) student participation, 7) involvement of parents and wider community (partnerships and networking), 8) sustainability, and 9) intersectoral working between the two key ministries of Health and Education, and the three additional thematic domains: 10) evidence-based support for the enablers and barriers discussed in the document; 11) specific considerations related to country income level; and, 12) specific considerations related to countries in humanitarian or fragile situations.

### Risk of bias and quality assessment

Because this was a scoping review, a formal risk-of-bias or critical appraisal assessment was not undertaken. The Melnyk and Fineout-Overholt Hierarchy of Evidence Grading Framework (2023) [8] was used descriptively to characterize the level/type of evidence represented by the included studies. This classification should not be interpreted as an assessment of methodological quality, risk of bias, or certainty of evidence.”

#### Synthesis and interpretation approach.

To review and evaluate this large body of literature consisting largely of descriptive studies and expert opinion and few research studies, a systematic approach involving thematic analysis with opportunities to interpret, synthesize, and validate findings was necessary. The following explicates the steps utilized to synthesize the data and develop themes for analysis and interpretation. Reviewers were assigned studies for data extraction. Relevant data from each included study were extracted using the 12 predefined categories. These categories served as the initial coding framework for data extraction. Relevant data from each included study were systematically extracted, often including direct quotations, summaries, or numerical data related to the research questions or objectives of the review. Following extraction, the research team compared findings across studies within and between categories using an iterative thematic analysis. Through discussion and consensus, related categories were refined and synthesized into broader themes that reflected patterns across the literature. Any differences in interpretation were resolved through reviewer discussion until consensus was achieved. Thematic analysis was used for identifying, analyzing, and reporting patterns (themes) within data. This involved coding the extracted data to identify recurring themes or patterns that emerged across the articles. Individual pieces of data were then assigned descriptive labels that captured the essence of the information. These labels were then grouped into broader themes based on similarities and connections. Themes were identified based on their frequency, significance, or relevance to the research question. Thematic analysis was iterative, with the researchers revisiting and refining themes as they analyzed the data more deeply. Meetings were held every two to four weeks to discuss thematic development. Once the themes were developed, the authors interpreted their meaning in relation to the constructs and the objective of the review. This involved considering the implications of the themes, exploring relationships between them, and identifying overarching patterns or concepts that emerged across the data. To enhance the rigor and trustworthiness of the synthesis and interpretation process, peer debriefing (consultation with colleagues) and triangulation (comparing findings across different sources or methods) were employed. Finally, the synthesized themes and interpretations were reported narratively, often accompanied by illustrative quotes or examples from the primary articles to support the findings. Articles identified in the updated search were extracted and coded using the same method and incorporated into the ongoing synthesis. The update review was completed by two reviewers (EM & DD). All reviewers contributed to the development of the most common enablers and barriers.

#### Publication bias.

Publication bias occurs when articles with statistically significant results are more likely to be published than those with null or nonsignificant findings, leading to an overestimation of effect sizes and potentially biased conclusions. This scoping review includes few research studies, as well as grey literature. As such, publication bias is more likely to occur through selective outcome reporting. We addressed this through three means: we used a comprehensive search strategy that aimed to include all relevant articles and documents from multiple databases, regardless of their publication status or language; we allowed the inclusion of unpublished (non-peer reviewed) documents such as dissertations, conference abstracts, and reports from government agencies or research organizations; and we directly assessed reporting bias through analysis of the included documents, such as selective outcome reporting or incomplete reporting of study methods.

## Results

### Demographic characteristics of peer reviewed and grey literature

Demographic characteristics of the included peer-reviewed and grey literature are presented in Table 1. Studies were obtained from all World Bank regions. The majority of documents described studies from North America (46%, n = 27), followed by Sub-Saharan Africa (27%, n = 16). World Bank income classifications were used to categorize the income level of the countries represented in the results. High-income countries (HIC) represented the largest percentage with 57% (n = 34) of the studies, upper middle-income countries (UMIC) were 18.6% (n = 11) of the studies and lower middle-income countries (LMIC) accounted for 18.6% (n = 11) of the studies. Low-income countries (LIC) represented 5.1% (n = 3) of the studies.

**Table 1:** Study country demographics by income level and literature type.

Country	n=	Percent	Region	Income	Lit Type
USA	23	38.9	North America	HIC	PR
USA	4	6.8	North America	HIC	Grey
Australia	2	3.4	East Asia & Pacific	HIC	PR
United Kingdom/ Europe	3	5.1	Europe & Central Asia	HIC	PR
Denmark	1	1.7	Europe & Central Asia	HIC	Grey
Japan	1	1.7	East Asia & Pacific	HIC	PR
International	3	5.1	Multiple	HIC/UMIC	Grey
China	1	1.7	East Asia & Pacific	UMIC	PR
Botswana	1	1.7	Sub-Saharan Africa	UMIC	PR
South Africa	5	8.5	Sub-Saharan Africa	UMIC	PR
South Africa	1	1.7	Sub-Saharan Africa	UMIC	Grey
India	3	5.1	South Asia	LMIC	PR
Pakistan	1	1.7	South Asia	LMIC	Grey
Ghana	1	1.7	Sub-Saharan Africa	LMIC	PR
Tanzania	2	3.4	Sub-Saharan Africa	LMIC	PR
Lao PDR	1	1.7	East Asia & Pacific	LMIC	PR
Nigeria	2	3.4	Sub-Saharan Africa	LMIC	PR
Nigeria	1	1.7	Sub-Saharan Africa	LMIC	Grey
Malawi	1	1.7	Sub-Saharan Africa	LIC	Grey
State of Eritrea	1	1.7	Sub-Saharan Africa	LIC	Grey
Sierra Leone	1	1.7	Sub-Saharan Africa	LIC	Grey
Total	59				PR = 45 Grey = 14

### Characteristics of the literature

Characteristics of the peer reviewed and grey literature are presented in S2 File. See Tables 2 & 3.

### Characteristics of the literature: level of evidence

Using the Melnyk & Fineout-Overholt (2023) Hierarchy of Evidence Grading Framework [8] for review of the literature, none of the included articles met the criteria for Levels 1–3 of the hierarchy, one article met the criteria for Level 4, one article met the criteria for Level 5, thirty articles met the criteria for Level 6 and thirteen articles met the criteria for Level 7. All of the grey literature documents met the criteria for Level 7. The level of evidence and source of overall themes are presented in Table 2.

**Table 2:** Level of evidence and source of overall themes.

Question	Theme	Level of Evidence*	Source of Evidence	
			Literature	Grey Evidence/ Policies
**1. Preparation and Planning for SHS:**	Enablers: Accessibility of health services, financial support, trauma-informed care, customized approaches, and baseline surveys facilitate effective preparation and planning for SHS.	Level 6=2 Level 7=8	9–13,17	14-16, 18
	Barriers: Managerial challenges, resource disparities, and poor data management hinder the preparation and planning process, impacting the effectiveness of SHS delivery.	Level 6=3 Level 7=3	19,21,24	20,22-23
**2. Policy and Institutional Anchoring of SHS:**	Enablers: National policy support, resource provision, capacity building initiatives, and inter-ministerial collaboration contribute to the institutional anchoring of SHS.	Level 4=1 Level 6=5 Level 7=10	13,21,24–28,30,32-33	22-23, 29,31,34-35
	Barriers: Lack of national policy coherence, unclear policy, challenges in policy implementation, and inadequate integration into the curriculum hinder standardization and prioritization of SHS initiatives.	Level 6=9 Level 7=1	25,36–44	N/A
**3. Learning and Management Practices for SHS Uptake and Sustainability:**	Enablers: Integrated health interventions, teacher training, health worker training, development of teaching aids, and collaborative approaches enhance the uptake and sustainability of SHS.	Level 4=1 Level 6=4 Level 7=7	11,27,33,44,45, 46,48,49	14,34,47,50
	Barriers: Data collection challenges, tracking students with disabilities, and insufficient teacher engagement hinder the effective implementation of SHS interventions.	Level 6=2 Level 7=3	27,36,44	20,23
**4. School Physical Environment and SHS Uptake:**	Enablers: Sanitary facilities, safe infrastructure, and conducive learning environments support the uptake and sustainability of SHS. Private spaces for confidentiality are required.	Level 6=4 Level 7=4	9,17,24,49,51	15,20,22
	Barriers: Resource insufficiency, challenges in access, and inadequate referral systems impede the delivery of SHS, particularly in underserved areas.	Level 6=1 Level 7=1	49	14
**5. School Social Environment and SHS Sustainability:**	Enablers: Integrated SHS-led health education, effective communication, and respectful learner autonomy foster a supportive social environment for SHS.	Level 5=1 Level 6=14 Level 7=7	11,19,24,27, 28,32,37,39, 40,43,45,48, 49,52-58	23
	Barriers: Violence, exploitation, and unsafe environments hinder student well-being and participation in SHS initiatives, necessitating comprehensive prevention strategies.	Level 4=1 Level 6=7 Level 7=1	13,24,25,33, 36,41,43,44	18
**6. Student Participation in SHS:**	Enablers: Adolescent-friendly resources, telehealth consultation, student representation, and strong referral systems promote student engagement and participation in SHS.	Level 5=1 Level 6=6 Level 7=1	19,39,42,49, 57,58,60	14
	Barriers: Lack of empowerment, limited access to counseling services, and inadequate operational improvements challenge student involvement in decision-making processes related to their health and well-being.	Level 6=4 Level 7=2	27,39,42, 49,59	23
**7. Parent and Wider Community Participation:**	Enablers: Induction and awareness meetings, public awareness campaigns, capacity building for schools, training community health nurses, empowering community members, involving school governing bodies, and offering flexible service provision facilitate the uptake and sustainability of SHS initiatives.	Level 6=5 Level 7=2	9,30,56,60,61	15,29
	Barriers: Include limited program awareness and internal referral processes, absence of establishment of SHS councils, not addressing cultural beliefs, and lack of formal partnerships with clear governance structures can impede efficient service delivery and undermine the effectiveness of SHS programs.	Level 6=3 Level 7=2	26,30,39,49	22
**8. Long-term Sustainability**	Enablers: Synchronization of implementation efforts, continuous capacity building facilitated by experienced personnel, training in planning and evaluation, comprehensive service provision covering various health needs, focus on behavior change interventions, resource allocation for both human and equipment needs, database management for effective resource utilization, trained school nurse leaders, specialized service provision through SHS mobiles and existing healthcare professionals, coordination between regional and national levels, consistent funding, and sector collaboration between health and education sectors contribute to the effectiveness and sustainability of SHS initiatives.	Level 4=1 Level 6=14 Level 7=7	10,12,13,19, 25,26,27,30, 32,33,37,38, 42,45,48,49, 53,54,55,56, 57	15
	Barriers: Inadequate resource allocation, lack of coordination and funding, low staffing levels, insufficient skilled school nursing staff, and insufficient collaboration between sectors can hinder the successful implementation and outcomes of SHS programs.	Level 6=7 Level 7=5	21,32,38–41,44,48,53,62,63	23
**9. Intersectoral Collaboration for SHS:**	Enablers: Training and referral systems, steering committee formations, regular coordination meetings, and involvement of multiple agencies enhance intersectoral collaboration for SHS.	Level 4=1 Level 6=7 Level 7=2	30,33,39,57,59,61,63,64	29,65
	Barriers: Insufficient awareness, unclear policy frameworks, and funding constraints pose challenges to sustained collaboration between the health and education sectors.	Level 6=6 Level 7=4	19,25,27,28,36,39,53,66, 67	23
**Levels of Evidence & Description** (adapted from Melnyk & Fineout-Overholt, 2023)^8^ Level 1: Evidence from a systematic review or meta-analysis of all relevant randomized controlled trials (RCTs) Level 2: Evidence from at least one well-designed RCT Level 3: Evidence from a single well-designed control trial without randomization (quasi-experimental studies) OR a systematic review of a complete body of evidence (integrative review of higher and lower evidence) OR mixed methods intervention studies Level 4: Evidence from well-designed case-control or cohort studies Level 5: Evidence from systematic reviews of descriptive and qualitative studies Level 6: Evidence from a single descriptive or qualitative study, evidence-based practice (EBP), evidence-based practice quality improvement (EBQI) and quality improvement (QI) projects Level 7: Evidence from the opinion of authorities and/or reports of expert committees, reports from committees of experts and narrative and literature reviews				

### Findings from the literature

The findings from the literature are described by the nine constructs aligned with the Health Promoting Schools framework; available evidence was too limited to support strong conclusions for the three additional constructs. See Table 5 in S3 File. Findings from the literature.

#### Preparing and planning for SHS.

Successful implementation of school health services (SHS) requires coordinated planning across school, district, and national levels. The literature identified several key enablers that supported effective SHS planning, including resource allocation, strong stakeholder collaboration, and the establishment of clear goals and guidelines [9–12]. Capacity building through targeted training and education strengthened local implementation efforts, while innovation and context-specific adaptation ensured relevance and sustainability [13]. Co-funded grants and access to free outpatient services [14,15], attention to trauma-informed care [16,17], and culturally tailored programming [18] have further supported equitable implementation.

However, systemic challenges presented major barriers. These include misaligned sectoral missions, incompatible funding structures, and inadequate communication across system levels [19]. Additional obstacles, such as limited staffing and funding, logistical challenges, and infrastructure deficits further impeded effective planning and execution [20]. Complex regulatory environments, short lead times, and lack of implementation-specific planning further complicated efforts [21]. Weak coordination between the health and education sectors, along with inequitable resource distribution was identified as a significant barrier to effective SHS planning and implementation [22,23].

While partnerships with the private sector offer potential for long-term sustainability, they introduce cost-effectiveness pressures that can influence service delivery priorities [24]. Ultimately, early planning, active stakeholder engagement, and systems-level alignment were essential to overcoming these barriers and achieving successful SHS implementation.

#### Policy and institutional anchoring of SHS.

One of the most important factors associated with ensuring policy and institutional anchoring of SHS was the design of national policy with specific reference to SHS priorities at both the national and local levels [21–25.] The literature noted that designers of health policy should consider stakeholder characteristics [13,26–28], incorporation of a common or shared vision across sectors, structures of policy enforcement, program evaluation, and financing.[29–31] Stakeholder engagement and organizational buy-in were enhanced when partners had an explicit understanding of program evaluation, whether through state mandates, program outcome reporting, or partner accountability [21,25,32,33]. At the school level, the use of a diverse group of stakeholders facilitated institutional anchoring by providing an environment where multiple perspectives and competing goals could be refined to advance well-structured, clearly outlined national policies. Government-led pilot programs and the integration of health into school curricula were also shown to promote institutional anchoring and long-term sustainability [34]. The integrated life-course approach to maternal, child and adolescent health service delivery described in the Eritrean Strategic Plan 2022–2026, is an example of how multigenerational health priorities can be integrated into SHS frameworks [35].

The most common barriers to ensuring policy and institutional anchoring were the lack of critical stakeholder support [36], lack of specific resource allocation, and the high-level priority changes encountered over time. Unclear or poorly constructed policy components led to a lack of consensus between stakeholders and perpetuated siloed decision making [25,37–40]. Lack of policy awareness, either through lack of wide-spread distribution and training or ineffective policy translation to practice also contributed to ineffective stakeholder support [39,41–44].

#### Learning and management practices.

Learning and management practices supported by legislation and policy enabled effective implementation of SHS [27]. Clear legislation provided guidance for school health professionals in the development of internal policy and practices and provide funding. Training and capacity building initiatives were more likely with legislation and local policies in place. With clear orientation, staff could effectively carry out the services and were more likely to continue with the implementation [44]. Collaborating with health professionals outside of the school enabled additional opportunities for training. Learning and management practices that included partnerships and collaboration with the community and organizations neighboring the school enabled the implementation of SHS [11,27,45] Support from these resources included funding, staffing and policy initiatives [46] For example, vaccine programs implemented in the school setting were successful when supported by local organizations and stakeholders such as parent groups and medical practices [33]. Non-profit governance boards and advisory groups are additional examples of supportive partnerships leading to the successful implementation of SHS. Training teachers to screen for common ailments and integrating health content into teacher education were effective strategies for strengthening early detection and sustainability [14,34]. Referral tools and training improved delivery [47].

Data management and monitoring are management practices that are vital to SHS success. Electronic systems were able to provide consistent monitoring which allowed for clear data collection and trending of information, providing data for effective service management [48].

Learning and management practices that adopted a holistic approach contributed to the successful implementation of SHS. Holistic approaches allowed for collaboration amongst health providers and educators, and encouraged family involvement in the SHS [49]. Successful holistic approaches also focused on engaging the community in addressing not only health but also social and educational needs within school settings [49]. A holistic approach can be taken within the structure of a templated business plan, designed to be a living document, that can be modified in response to unique community needs [50].

Many of the enablers, when absent, were noted as learning and management practice barriers. Access to medical records from settings outside of the school was a hindrance that led to a lack of coordination between the SHS and health professionals outside the setting, contributing to the inability to effectively carry out SHS [44]. Failure to gain support from stakeholders such as community members, families and the school administration was often noted as a barrier to the implementation of SHS [27,36]. Additionally, inconsistent data collection practices and limited tracking of students with disabilities remain significant barriers to effective learning and management practices in SHS [23]. However, the greatest barriers to effective implementation of SHS were resource constraints and subsequent workload management [20]. Without appropriate funding and staffing, SHS were not able to succeed.

#### School physical environment.

The ability to have a private area with sufficient space to provide SHS was vital [49]. Safety was also noted as important and included a safe space to provide services along with enough safe storage space for equipment and supplies. Locating services on or adjacent to school grounds helped to facilitate the success of SHS as it increased the likelihood of students attending appointments, minimized the time they spent away from the classroom, and reduced demands on parents [24]. This was found to have particular importance for children living in poverty [17]. Integration and accessibility were also noted as enablers to the implementation of SHS. Space to hold group sessions and access to telehealth equipment increased the availability of SHS. Clear funding streams to provide space for health services, access to necessary healthcare supplies like first aid kits and emergency medications, as well as access to healthcare professionals to provide SHS are additional enablers noted for the school physical space [20,51]. Functional sanitation facilities, clean water, and safe infrastructure have been identified as critical enablers of SHS delivery, supporting both health and learning outcomes [9,15,22].

Barriers included lack of physical space and spaces seen as safe, providing both confidentiality and physical safety for the students [49]. Major barriers also included insufficient staff, inadequate equipment, and limited transportation options, which disproportionately affected schools in remote areas [14].

#### School social environment.

The most important enablers of successful SHS implementation were collaboration and partnership among healthcare providers, school personnel, students and the broader community [24,39,40,43]. These partnerships were most effective when they included clearly defined roles, flexibility, open communication and shared goals [28,49,52]. A shared understanding that student health is essential for academic success was a foundational component of successful SHS [37,53].

Implementing SHS often required new interdisciplinary and intersectoral collaborations that necessitated the reorganization of existing roles [54]. The most successful models involved active participation by health and education stakeholders in shaping their responsibilities [48,54]. This redesign was most effective when supported by organizational readiness, thoughtful role planning, targeted training, and mechanisms for continuous communication and relationship building [19,28,55–57]. Nearly all sources emphasized that ongoing effective communication is critical to fostering a positive school social environment [27,32,55,58]. Adequate workload infrastructure also supported communication, which was often overlooked when staff were overburdened. To support sustainability, infrastructure support to enable manageable workloads was essential so that new responsibilities did not merely add to existing roles [11,32,45,54]. Conversely, lack of effective collaboration among health professionals, school staff, students, and community members was a key barrier to SHS implementation. Reasons for lack of collaboration included lack of a shared understanding of the role of health in education, competing priorities, and workload constraints that made it difficult to incorporate health-related responsibilities [25,43,44].

Creating safe and inclusive environments free from violence and abuse, with enforced codes of conduct, was shown to enhance school climate and improve SHS outcomes [23]. Cultural mismatches and lack of trust between school personnel and the local community served as significant barriers to SHS uptake, despite best intentions toward inclusion [18]. Even in diverse communities, conflicting cultural values between health professionals and families obstructed engagement with SHS [24]. Broader social conditions, such as extreme poverty and weak infrastructure, further hindered SHS development [36,41]. In high-income countries, inequities stemming from intersectoral social factors may create barriers to fair and consistent SHS access [13,33].

#### Student participation.

Students’ purposeful and active engagement in the development, implementation and evaluation of SHS was considered to be an important principle in the uptake of SHS, yet few sources indicated this to be a widely implemented practice [39,42,49]. Most often, the planning, implementation, and evaluation of SHS was carried out with extensive planning for the students, rather than with the students [39]. While it was seen as best practice to systematically collect feedback from students to assess needs or evaluate their experience to inform the provision of health services and initiatives of the SHS, student input was infrequently sought [42,49]. It was noted that even when students were involved locally in some aspect of planning, implementation and evaluation of SHS, students were typically not involved in higher levels of administration of the services, such as in legislation or monitoring [27,42,59]. When students were involved in the initial phases of implementation of SHS, consistent and sustained systems for student input were not in place, and student input diminished over time. Lack of supportive infrastructure and heavy workloads for both educational staff and healthcare providers was cited as precluding the ability to actively engage students in this manner [49]. However, there were examples of successful student involvement through several means, such as active identification of student needs through surveys [19,60], identification of students who could serve as health promoters/educators and asked for informed feedback, and the implementation of student advisory groups [19,42,57]. Examples of successful engagement include the use of representative student councils and school health clubs, which fostered ownership and enhanced the sustainability of SHS initiatives [14], and mental health services designed for student choice within a clear delivery structure [58]. Nonetheless, the absence of youth representation in decision-making processes and inconsistent referral systems continue to act as barriers to meaningful student participation in SHS [23].

#### Involvement of parents and wider community.

Effective collaboration between parents and schools is essential; when parents engage in health committees, communicate with educators, and promote healthy habits at home, SHS programs thrive [9,29,61]. Schools that establish strong partnerships with local organizations and health services further enhance the success of these programs. Providing free, on-site health support made it more convenient for parents to participate, while granting schools autonomy over their health initiatives fostered a sense of ownership and dedication. Clear and consistent communication and education through meetings, media, and technology also boosted parental [30] and community [60] involvement. Training schools in fund management and equipping community health nurses were identified as effective strategies to enhance parental engagement and service accessibility in SHS [15].

However, parents could also be perceived as a challenge to progress [30,39]. Parental disagreement with school policies related to sexual health led to resistance [49]. Language barriers made it difficult for non-English-speaking families to access services. Misinformation and fear of health interventions, such as vaccines, resulted in reluctance to utilize available services. Complex enrollment procedures and consent forms deterred parents from signing up their children [49]. Expanding health programs sometimes led to operational issues like longer wait times, causing frustration among parents. Excluding school governing bodies and community stakeholders during the planning phases was also cited as a barrier that undermined trust and limited long-term SHS sustainability [22,26]. Addressing these issues required actively listening to community concerns and involving parents, thereby building trust and strengthening school health programs [56].

#### Sustainability.

Ensuring the long-term sustainability of SHS required systemic commitment at the national level, supported by reliable resources and strong intersectoral partnerships [10]. At the policy and planning level, sustainability hinged on stable financing structures, multisectoral alignment, and collaborative partnerships that were built on transparency, shared vision, and effective communication [10,12,25,27,30,32,33,37,42,48,53,54,56], National programs that are rooted in local data and embedded within continuous quality improvement frameworks were more likely to remain relevant and effective over time [13,26,30,38,49,55,57]. Furthermore, delivering a comprehensive SHS package, including preventive care and behavior change interventions, was shown to support long-term impact and responsiveness to evolving student health needs [15].

Despite its importance, sustainability remains challenging to achieve. Many programs rely on short-term grants, inconsistent public funding, or sector-specific billing systems that complicate implementation and continuity [21,44,48,62,63]. Inadequate coordination between national and regional structures, as well as the absence of robust data systems, undermined scalability and long-term planning [23]. A critical constraint across nearly all contexts is the availability of qualified personnel, particularly nurses. Persistent nursing shortages, high costs, and lack of BSN (university) prepared or trained school nurses limit both capacity and program expansion [32,38–41,53,63].

At the operational level, sustainability was strengthened by investing in local infrastructure and human resources within schools [45]. Establishing school-based health teams, such as those led by nurses supported service continuity while building local leadership capacity [32]. School-based telehealth programs offered scalable models for expanding access, especially when embedded within broader partnerships [55]. The purposeful engagement of diverse stakeholders in school-level planning fostered collaborative problem-solving and shared ownership, both of which were critical to sustaining SHS at the point of care [19].

#### Intersectoral working between the two key ministries of health and education.

Effective intersectoral collaboration between the Ministry of Health (MOH) and the Ministry of Education (MOE) was a foundational enabler for sustainable SHS. Legislation played a key role in aligning national priorities, whether targeting specific issues like immunization [33] or supporting comprehensive SHS, as demonstrated by Japan’s longstanding national SHS framework [64]. Sierra Leone’s School Health Policy offers a detailed blueprint for intersectoral collaboration that exemplifies actionable strategies for expanding SHS in low-income contexts [65].

Legislation must be well-aligned across ministries and accompanied by active government involvement to avoid regulatory confusion, legal concerns, and gaps in implementation [19,28]. Misalignment between sectors and limited government engagement have been shown to undermine coordinated efforts and accountability; strong collaboration among government bodies, schools and international partners drives success [29].

Differences in organizational culture and ideology between the health and education sectors often challenged collaboration [36,66]. These tensions required inclusive leadership approaches that fostered mutual understanding, built capacity, and drove change [30,63]. Leadership may be shared across sectors or vested in dedicated task forces or national steering bodies [57,61], but consistent communication, common goals, and clarity of purpose remain essential [29,39]. Weak coordination between national and regional entities, and inconsistent stakeholder engagement limited the effectiveness of joint SHS delivery [23,67]. Structural fragmentation, lack of planning, and disengaged stakeholders further contributed to ineffective collaboration [25,27,39,53].

At the school level, intersectoral coordination was strengthened through strategic resource allocation and human resource planning. The addition of school nurses as institutional personnel, alongside sustainable systems for training and deployment, supported the delivery of core SHS functions. Task forces that connected schools with both MOH and MOE structures enabled local problem-solving and implementation oversight. Partnerships that provided external funding or staffing, such as clinical placements for student nurses or nurse practitioner interns, also enhanced capacity and continuity [33,59]. These multi-level strategies were essential for operationalizing national mandates and ensuring that intersectoral collaboration reached the point of care within schools.

## Discussion

This study presents a scoping review of peer-reviewed literature alongside a synthesis of grey literature to examine the barriers and facilitators influencing the implementation of SHS. The peer-reviewed literature emphasizes the importance of intersectoral collaboration grounded in a shared vision that integrates health and education. Schools play a critical role in promoting health, and their involvement is essential to successful SHS implementation. Clear, evidence-informed legislation that aligns national, subregional, and local policies, while accounting for diverse cultural, contextual, and resource environments, is a key enabler. Effective policies established environmental standards for SHS spaces, outlined resource requirements, and defined clinical competencies for SHS personnel.

Sustainable SHS implementation depended on robust funding mechanisms. Sources of funding included governmental programs, academic-practice partnerships, and innovative business models. Funding that supported cross-sector data documentation and management systems enhanced collaboration; its absence presented a significant barrier. Capacity building must encompass workforce development for new and evolving roles, not only for the SHS workforce, but also for educators and support staff. Ongoing communication and training systems were necessary to address local challenges in real time.

Stakeholder engagement was vital. This included students, families, communities, and political actors. A failure to recognize and engage these groups derailed implementation efforts at all levels. Ultimately, a shared vision embedded in policy, supported by intersectoral collaboration, adequate funding, and comprehensive capacity development, including workforce readiness and stakeholder systems, was essential for successful SHS delivery.

The grey literature synthesis reinforced these findings, highlighting that sustainable SHS requires strong policy frameworks, intersectoral collaboration, and active community participation. Key enablers included national education policies, trauma-informed practices, integrated health education, and the presence of dedicated school health personnel. Success was further driven by teacher and student engagement, adequate physical infrastructure, flexible and responsive service delivery, and data-informed planning. Conversely, common barriers included fragmented coordination, insufficient resources, underdeveloped data systems, and the absence of cohesive national policies. Long-term implementation depended on sustained capacity building, collaborative governance, and inclusive participation from students, families, and relevant ministries. A holistic, equity-driven approach rooted in behavior change and community partnerships underpins the lasting impact and resilience of SHS initiatives.

### Strengths and limitations

The level of evidence of the literature limits the strength of these findings; however, the consistency of the findings across this broad search of literature adds credence to the body of the published works. There is a considerable risk of publication bias in both the grey and peer-reviewed literature; the grey literature lacks scientific review and the peer-reviewed literature is largely descriptive, with both subject to the tendency to publish only positive results. There is also considerable heterogeneity of SHS models across settings, along with a lack of standardized implementation outcomes across studies. Nonetheless, the Health Promoting Schools framework provided a robust structure for eliciting descriptions of effective implementation strategies across settings and studies. Despite efforts to obtain studies of sustainable programs, most of the literature focused on new implementations or specific interventions. Much of the literature indicated a lack of formal evaluation of programs, which could have given additional insight into this review. As some articles noted, SHS are often siloed and there is a lack of tracking and data collection on a regular basis, which contributes to the lack of more strongly designed studies. The literature may also not be globally representative. Although a wide range of databases were searched with no restrictions on publication language, the search terms used were in English only, which limited the ability to identify non-English language publications, and relied on artificial intelligence built within the databases. As a result, relevant publications in other languages may not have been identified, potentially affecting the comprehensiveness of this review. Although efforts were made to search the literature from all countries, most of the literature was from high income countries, and many countries and regions of the world were not represented. However, some articles originating in high income countries described SHS in countries across all income levels. To advance understanding of best practices for implementing effective SHS, future publications should prioritize intentional evaluation of successful SHS programs across all countries.

## Conclusion

There is compelling descriptive evidence and low-level research evidence to support intersectoral collaboration, policy coherence, stakeholder engagement, sufficient funding, and workforce capacity building as crucial factors for successful SHS implementation. The findings support several key enablers. Intersectoral collaboration grounded in a shared vision that integrated health and education was critical. Strong policy frameworks aligning policy at all levels while accounting for diverse cultural, contextual and resource environments were key to providing necessary structure. Stakeholder engagement that included health providers, school administration, educators and school staff, parents, students and the wider community contributed to meaningful health programs that addressed local needs. Robust funding mechanisms were needed to support sufficient workforce and adequate physical infrastructure. Workforce capacity building was necessary to support new roles and address local challenges in real time. To advance understanding of best practices for implementing SHS, intentional evaluation of successful SHS programs in all countries is needed for future publications. These results provide policy makers, practitioners and other stakeholders with a rich description of strategies that have supported successful implementation of SHS globally.

## Supporting information

S1 File Search strategy. (DOCX)

S2 File Tables 2&3. Characteristics of the literature. (DOCX)

S3 File Table 5. Findings from the literature. (DOCX)

S4 File PRISMA-ScR checklist. (DOCX)
