# Supplementary material for: Enablers of and barriers to implementation of school health services: A scoping review
Source: PLoS One. 2026 Nov 13;21(11):e0361415. doi: 10.1371/journal.pone.0361415 (PMC13766220; doi:10.1371/journal.pone.0361415)
Supplement: Supplementary file 1 [file pone.0361415.s001.docx]

**S1 Text. Search Strategy**

**Implementation of School Health Services (SHS)**

**PubMed**

(((Plan*[Title/Abstract] OR implement*[Title/Abstract] OR prepar*[Title/Abstract] OR steps[Title/Abstract] OR administration[Title/Abstract] OR sustain*[Title/Abstract] OR success*[Title/Abstract] OR effectiv*[Title/Abstract] OR efficacy[Title/Abstract] OR performance[Title/Abstract] OR assess*[Title/Abstract] OR evaluat*[Title/Abstract] OR monitor*[Title/Abstract] OR analysis[Title/Abstract] OR analy*[Title/Abstract] OR outcome[Title/Abstract] OR result*[Title/Abstract] OR Policy[Title/Abstract] OR policies[Title/Abstract] OR procedures[Title/Abstract] OR practices[Title/Abstract] OR "physical environment"[Title/Abstract] OR "social environment"[Title/Abstract] OR participation[Title/Abstract] OR enablers[Title/Abstract] OR Facilitators[Title/Abstract] OR challenges[Title/Abstract] OR barriers[Title/Abstract]) OR ("program development"[MeSH Terms] OR "program evaluation"[MeSH Terms])) AND ((("nurs*"[Title/Abstract] OR "health* professional"[Title/Abstract] OR "health provider"[Title/Abstract] OR dentist[Title/Abstract] OR dental[Title/Abstract] OR physician[Title/Abstract] OR doctor[Title/Abstract] OR medical[Title/Abstract] OR psychologist[Title/Abstract] OR "school district*"[Title/Abstract] OR "ministry of health"[Title/Abstract] OR government[Title/Abstract]) OR ("health personnel"[MeSH Terms])) AND ((( "school health services"[Title/Abstract] OR "school health program*"[Title/Abstract] OR "school-based health*"[Title/Abstract] OR "school located immunization"[Title/Abstract] OR "School dental clinic"[Title/Abstract] OR "school dental program*"[Title/Abstract] OR "school clinic"[Title/Abstract] OR "School-based clinic"[Title/Abstract] OR "School health centers"[Title/Abstract] OR "school health centres"[Title/Abstract] OR "school-based primary care"[Title/Abstract] OR "school nurs*"[Title/Abstract]) OR ((("school health services"[MeSH Terms]) OR ("school mental health services"[MeSH Terms])) OR ("school nursing"[MeSH Terms]))))) AND ((( "school health services"[Title/Abstract] OR "school health program*"[Title/Abstract] OR "school-based health*"[Title/Abstract] OR "school located immunization"[Title/Abstract] OR "School dental clinic"[Title/Abstract] OR "school dental program*"[Title/Abstract] OR "school clinic"[Title/Abstract] OR "School-based clinic"[Title/Abstract] OR "School health centers"[Title/Abstract] OR "school health centres"[Title/Abstract] OR "school-based primary care"[Title/Abstract] OR "school nurs*"[Title/Abstract]) OR ((("school health services"[MeSH Terms]) OR ("school mental health services"[MeSH Terms])) OR ("school nursing"[MeSH Terms])))

**CINAHL**

S1 ( "school health services" OR "school health program*" OR “school-based health*" OR “school located immunization” OR “School dental clinic” OR “school dental program*” OR "school clinic" OR “School-based clinic” OR “School health centers” OR “school health centres” OR “school-based primary care”  ) OR (MH "School Health Services+") OR (MH "School Mental Health Services") OR (MH "School Nursing") OR (MH "School Nurses")

S2 ("nurs*"OR “health* professional” OR “health provider” OR dentist OR dental OR physician OR doctor OR medical OR psychologist OR "school district*" or "ministry of health" or government) OR (MH "Health Personnel+")

 S3 ( Plan* OR implement* OR prepar* OR steps OR administration OR sustain* OR success* OR effectiv* OR efficacy OR performance OR assess* OR evaluat* OR monitor* OR analysis OR analy* OR outcome OR result* OR Policy OR policies OR procedures or practices OR “physical environment” OR “social environment” OR participation OR enablers OR Facilitators OR challenges OR barriers) OR (MH "Program Development+") OR (MH "Program Evaluation") OR (MH "Government Programs+")

S4 S1 AND S2 AND S3

**ERIC, Global Health, Web of Science,**

S1 ( "school health services" OR "school health program*" OR “school-based health*" OR “school located immunization” OR “School dental clinic” OR “school dental program*” OR "school clinic" OR “School-based clinic” OR “School health centers” OR “school health centres” OR “school-based primary care”  OR “school nurs*”)

S2 ("nurs*"OR “health* professional” OR “health provider” OR dentist OR dental OR physician OR doctor OR medical OR psychologist OR "school district*" or "ministry of health" or government)

S3  ( Plan* OR implement* OR prepar* OR steps OR administration OR sustain* OR success* OR effectiv* OR efficacy OR performance OR assess* OR evaluat* OR monitor* OR analysis OR analy* OR outcome OR result* OR Policy OR policies OR procedures or practices OR “physical environment” OR “social environment” OR participation OR enablers OR Facilitators OR challenges OR barriers)

S4 S1 AND S2 AND S3

( ( "school health services" OR "school health program*" OR “school-based health*" OR “school located immunization” OR “School dental clinic” OR “school dental program*” OR "school clinic" OR “School-based clinic” OR “School health centers” OR “school health centres” OR “school-based primary care”  OR “school nurs*”) ) AND ( ("nurs*"OR “health* professional” OR “health provider” OR dentist OR dental OR physician OR doctor OR medical OR psychologist OR "school district*" or "ministry of health" or government) ) AND ( ( Plan* OR implement* OR prepar* OR steps OR administration OR sustain* OR success* OR effectiv* OR efficacy OR performance OR assess* OR evaluat* OR monitor* OR analysis OR analy* OR outcome OR result* OR Policy OR policies OR procedures or practices OR “physical environment” OR “social environment” OR participation OR enablers OR Facilitators OR challenges OR barriers) )

**LILACS**

school health services AND implementation AND ( db:("LILACS")) AND (year_cluster:[2000 TO 2023 and 2023-2025])

[Portuguese (179)](javascript:%20add_filter('la_fc9fdf084e290f26a270390dc49061a2');)

[Spanish (53)](javascript:%20add_filter('la_12470fe406d44017d96eab37dd65fc14');)

[English (47)](javascript:%20add_filter('la_9cfefed8fb9497baa5cd519d7d2bb5d7');)

**Cochrane Central Trials**

(school NEXT health NEXT services) OR (school NEXT health NEXT program*) OR (school-based NEXT health*) OR (school NEXT located NEXT immunization) OR (School NEXT dental NEXT clinic) OR (school NEXT dental NEXT program*) OR (school NEXT clinic) OR (School-based NEXT clinic) OR (School NEXT health NEXT centers) OR (school NEXT health NEXT centres) OR (school-based NEXT primary NEXT care) OR (school NEXT nurs*) in Title Abstract Keyword AND (school NEXT district*) OR (ministry NEXT health) OR government OR nurs* OR (health NEXT professional) OR (health NEXT provider) OR dentist OR dental OR physician OR doctor OR medical in Title Abstract Keyword AND Plan* OR implement* OR prepar* OR steps OR administration OR sustain* OR success* OR effectiv* OR efficacy OR performance OR assess* OR evaluat* OR monitor* OR analysis OR analy* OR outcome OR result* OR Policy OR policies OR procedures or practices OR “physical environment” OR “social environment” OR participation OR enablers OR Facilitators OR challenges OR barriers in Title Abstract Keyword - with Publication Year from 2000 to 2023 and 2023-2025, with Cochrane Library publication date Between Jan 2000 and Oct 2023 and Aug 2025 in Trials
