# Supplementary material for: Enablers of and barriers to implementation of school health services: A scoping review
Source: PLoS One. 2026 Nov 13;21(11):e0361415. doi: 10.1371/journal.pone.0361415 (PMC13766220; doi:10.1371/journal.pone.0361415)
Supplement: Supplementary file 2 [file pone.0361415.s002.docx]

**Table 2**. **Characteristics of included peer-reviewed literature**

| **First author; (year); country** | **Description include level of implementation (e.g. School, school district, national, etc.), mandated status, generalizability, number of schools/districts, demographics, time frame.** | **Framework; program** | **Health target; specific focus (research aims)** | **Schooling level; schooling type** | **Research methods (study type, respondents, data type, data collection)** | **No. of respondents; Type of respondent** |
| --- | --- | --- | --- | --- | --- | --- |
| Adams, (2000); US | School district, 1 school nurse per 6000. | Grant to support clinical sites in two schools. | 1) Fill gaps in school health program 2) Legislation for school nurse positions 3) Faculty needed for preparing new graduates for school nurse practice. | Rural public elementary (primary) schools | Qualitative. Two elementary schools and regional health center to promote healthy outcomes and basic health services delivery. Based on 3 national educational goals. | 6000 students in a 12-school district with one school nurse graduated from school 6 months ago.  1. Primary 536 children, 61% low income.  2. Primary 652 children, 74% low income. |
| Ademokun (2014); Nigeria | Principals of schools in the urban local government. Sampling frames for all the schools in each local government were obtained and proportional allocation | Key Informant Interview was utilized | 1) Children suffer from anemia and therefore potential for low cognitive ability 2) Education and health Millennium Development  3) Achievement of universal primary education; 4) Reduction of child mortality; and  5) Combatting HIV/AIDs, malaria and other diseases, significantly. | 21 schools serving ages 6-14 | Qualitative: Key informant Interview guides and ‘Observational checklists’.  1-Healthful School Environment 2-School Health Services 3-Skill based health education 4-School Feeding Services | Principals of each school interviewed. |
| Adomako Gyasi (2023); Ghana | 123 primary schools in Ejisu Municipality in Ghana’s Ashanti region | 2030 Agenda for Sustainable Health Development Goals; Theory of Planned Behavior | Identification of barriers hindering the implementation of School-based health programs in Ghana | Primary schools | Qualitative study, focus group interviews with stakeholders, self-report, interview guide, audio recording, transcribed with Nvivo software, thematically analyzed, informed consent | 116 stakeholder participants involved in the implementation of health programs |
| Ahmed (2023); South Africa (SA) | Single health sub-district within the Western Cape, SA, not mandated, low generalizability, secondary schools, 2 semester implementation period | “standard package of healthcare services” pilot implementation | Delivery of general wellness, Pregnancy, sexually transmitted illness (STI) and Tuberculosis (TB) screening, promotive health (substance use, mental health) sexual health services (HIV testing, contraceptive counseling) | Secondary schools | Descriptive study, data collected: % of visits, choice of service | Report of service utilization of 344 students over 42 schools |
| Association of Immunization Managers, National Association of School Nurses (2022); US | National; five virtual roundtables (focus groups) with 15 school nurses and 15 state immunization program managers from across the United States; solicited information about participants’ experiences planning and implementing School located vaccine clinics (SLVs)for COVID-19 and influenza, factors that influence SLV success, and resources to implement SLVs. | school-located vaccination clinics (SLVs) | influenza immunization; The objective of the roundtables was to build on findings from the environmental scan of school-located vaccination models to further explore key challenges and opportunities for planning and implementing of same | Primary and secondary schools from urban and rural geographies | five virtual roundtables with 15 school nurses and 15 state immunization program managers from across the United States: Mid-Atlantic (3), Midwest (9), Mountain/Central (5), New England (3), Pacific (3), Southeast (7) | 15 school nurses and 15 state immunization program managers from across the United States |
| Baptiste (2005); United Kingdom | Expanding school nurse teams including healthcare assistants (HCA). | SHS promotion by expanding nursing and medical workforce. | Team approach in school nursing is possible success dependent on  1) developing equitable workloads,  2) team leadership,  3) development training 4) effective communication. | Primary and secondary schools. Each nurse manages 4-5 primary schools or 1-2 secondary. The pilot included 3 teams, the nursing teams, the school health doctors and the education staff. | Qualitative. clustering simple categories utilizing a coding system. focus group discussions and semi structured interviews. | nurse=5, HCA = 4 groups. Interviews and discussions in primary and secondary schools. |
| Bosede (2023); Nigeria | Study to assess implementation of the School Health Program (SHP) in Ondo state of Nigeria. | Promoting the SHP in all schools. | Assess the implementation of the five components of SHP as described in the National School Health Policy (NSHPo) | Primary and secondary. | Descriptive cross-sectional study. Used an observational checklist. | 42 schools, 33 from urban district and 9 from rural district. |
| Boyer-Chu (2013); US | Implementation of additional dose of Tdap for grades 7-12 (secondary) | Collaboration with public health and school district | By the first day of the 2011–2012 school year, students in 7th through 12th grades either provide proof of having received Tdap or provide a medical or personal waiver. | grades 7-12 (secondary) | Qualitative. Data gathering from Centers for Disease Control) (CDC), local Public Health | 23,300 student grades 7-12 (secondary) |
| Burman (2023); Australia | Semi-structured qualitative interviews. One school |  | Examine opinions of stakeholders to gain insight into important considerations for model, to guide the “Our Mia Mia” program's evolution and shape the developing of similar emerging health hubs | Primary | Descriptive study: One to one interviews 30-45 min Children's interview = 4-5 in a group with caregivers one hour Semi structured interview guide, with conversation allowed to flow organically; themes included participant involvement with "Our Mia Mia", their overall perceptions, elements they felt were successful, elements they felt apprehensive about, challenges perceived, vision for the future Transcripts analyzed using grounded theory approach; emergent coding of themes, theory formulation until saturation | N=55 School principals 6 Director of Education 1 school directors 2 school manager 1 teachers 13 school psychologists 6 parents 7 students 5 health and social service providers 11 Aboriginal Education Officer 1 Aboriginal Education Consultants 2 Aboriginal agency staff 1 |
| Daly (2011); US | High (secondary) school students in a small New England city-use of a School Based Health Center (SBHC) to provide sexual health services to adolescents | Transtheoretical Model | Use of School Based Health Centers to provide contraceptive services to teens | High (secondary) schools | Qualitative | High (secondary) school students in New England |
| Emond (2023); US | Elementary (primary) schools-implementation and challenges of school-based health care clinics | Delphi Methodology | The purpose of this study was to better understand the leadership challenges when developing SBHCs, add context to the problem, and share the findings in order to create a comprehensive collaborative leadership model. | Elementary (primary) schools | Qualitative | 20 school superintendents, medical sponsor administrators, SBHC administrators, and community mental health directors |
| Glow (2003); US | 7 Academic Partnership Centers-Rush University College of Nursing provided health services with their nurse practitioner students to local Chicago public schools | Community Collaborative Partnership | Implementation and effectiveness of health services through academic partnership centers | Middle/high school (ages 13-16) (secondary schools) | Quasi-experimental, collaboration between Chicago public schools and Rush University School of Nursing | 1306 students ages 13-16 |
| Hayter (2021); United Kingdom | National, non-mandated, phone interviews with individuals identified as having a lead role in the development of School Based Health Services (SBSHS) across the UK. Limited generalizability |  | Aim: Explore the experiences of clinicians/managers in developing and delivering school based sexual health clinics. 1. What are the barriers to establishing school-based sexual health clinics? 2. What factors facilitated effective working in this area? 3. What were their experiences of how services were developed and evaluated? | Not specified. | Descriptive study: Qualitative: UK mapping survey; semi structured phone interviews with individuals identified as having a lead role in the development of SBSHS across the UK | N=51 Teenage pregnancy coordinator = 13 Clinical staff with service management roles (school nurses and school medical staff =12 Clinical managers with responsibility for children and young people's school health services =10 Coordinators of local sexual health service or project = 8 External consultant on school sexual health = 2 |
| Howland (2018); US | Multiple schools in the state of MA Focus group = limited generalizability |  | Assess implementation of legislated regulations relative to the management of students' head injuries occurred during extracurricular sports | High (secondary) school | Descriptive study: Focus group with school nurses, athletic trainers | N=21 School nurses = 11 Athletic trainers = 10 |
| Jakasania (2023); India | Literature review of School Health Program (SHP) through government initiatives in India, review of other LMIC | Health Promoting Schools; Ayushman Bharat program, Focusing Resources on Effective School Health (FRESH) (UNESCO, United Nations Children’s Fund (UNICEF), WHO) | Identification of status of school health programs in India, gap analysis | Primary and secondary schools | Literature review, expert recommendation | N/A |
| Jain (2022); India | Interviews with key informants, manuals and operational guidelines reviewed, limited generalizability | WHO Universal Health Coverage (UHC) | 1. Aim is to provide a comprehensive overview of adolescent health programs in India with a focus on strengths and limitations in the planning and implementation* Note: this article covers all adolescent health programs, and examples that are given are focused on health education, and teacher implementation, though "comprehensive" health services are referred to. The authors note that informants were all at the national level, and not at the local level. However, this article does focus on barriers and is a good support for the other articles that focus on facilitators. | Adolescent school health | Descriptive study: Semi- structured interviews and desk review based on WHO tool for assessing the implementation of adolescent and SHS programs, 50-60 minutes. Data analyzed using WHO framework for UHC for adolescents. | N=18 senior government officials 8 (4 health, 4 education) private civil service org officials 8 clinician 1 technical support 1 |
| Jenlink (2010); US | Report: Case study, multiple school districts across several states, generalizability may be related to the use of established protocols (Incident Command), resources (CDC and federal government) |  | Report of facilitators and barriers encountered in setting up school-based vaccination clinics for H1N1 | Elementary and high schools (primary and secondary schools) | Interviews with vaccination clinic planners | Not specified |
| Jennings (2020); US | Case study report |  | Describe the implementation of 10 strategically located Youth and Family Centers (YFC) | Elementary, middle and high schools (primary and secondary schools) | Case study report by medical, educational and service leaders |  |
| Johnson (2020); US | Evaluation of implementation strategies of SBHCs in 3 disparate communities |  | Evaluate factors r/t implementation and sustainability of 3 school-based health centers | Elementary, middle and high schools (primary and secondary schools) | Descriptive study: Mixed methods: Quantitative data on student enrollment, utilization, health outcomes, seat time, patient revenues Qualitative data via key informant interviews 2-year period |  |
| Jones (2019); Australia | Region - rural New South Wales; population includes Indigenous people |  | Primary Healthcare Registered Nurse: School Based (PHCRN:SB) strategy and implementation; Obj 1: implementation factors for strategy designed to address the health, education, and social inequities of rural children and adolescents; Obj 2: investigate facilitators of strategy targeting cross-sector collaboration, relationships, resources, and workforce. | Primary and secondary schools | Qualitative, descriptive paper of early stages of PHCRN:SB strategy implementation; |  |
| Lankford (2019); US | Schools in 18 counties of rural Alabama; limited generalizability; services through medical order only. | Whole Child Framework | Occupational therapy direct services; | Public school, special education population as related service | Descriptive overview of program; generalized |  |
| Lear (2002); US | State level, school district; Identify enablers and barriers to SHS reorganization in two different US states, in response to goal of national single system of healthcare. limited generalizability |  | Objective 1: examine two level of strategies to reorganize and enhance quality of SHS. Objective 2: examine the solutions proposed by each control level to the problems described. | Primary and secondary schools. Public. | Descriptive, 2 scenarios discussed: State level, community/ district level, two different states. |  |
| Li (2023); China | 10 primary schools in Henan Province, interviews conducted over 2.5 years (3/20-8/22) concurrent with implementation of evidence-based mental health services in 10 schools | Multilevel model (EBP subject to macro, school and individual level factors) | Mental health services; Aim: explore factors that affect implementation of EBP in schools and provide practical advice to promote their development | Primary schools | Qualitative: thematic analysis of semi-structured interviews of participants who worked in schools | 26 respondents; 4 education bureau leaders, 8 school administrators, 7 classroom teachers, 7 service implementers |
| Mackert (2007), US | 31 schools in Kansas  project of Kansas University Medical Center (KUMC) Case study = limited generalizability Descriptive study |  | Investigate critical factors of success in TeleKidcare a school-based telemedicine project  RQ1a: What are key steps to be taken in the planning of a telemedicine system?  RQ1B: What individuals should be involved in planning?  RQ2: What factors contributed to the success of a multi-site telemedicine system?  RQ4: What contributed to the complete adoption and continued operation of the system beyond the initial research phase of operation? |  | Case study. Interviews with project documentation to corroborate the interview data. | N=23 TeleKidcare administrators: 5 KUMC providers: 6 School nurses: 12 |
| Maki (2019); US | Boston School District, 10 schools, 4-month period post-1 yr. implementation; limited generalizability | Comprehensive Behavioral Health Model | Mental health; Obj 1: examine the knowledge attitudes, and beliefs of school psychologists during the early implementation of expanded professional role, including support and barriers involved Obj2: inform systems-change literature to enhance future rollout, provide recommendations | Primary and secondary public schools | Qualitative, semi structured individual interviews, voluntary participation | n= 10: Primary and secondary school psychologists |
| Mason (2019); US | Regional (city) - New York City. Identify implementation of Family Health Center Model for school-based oral health programs in NYC. generalizable to similar programs | Family Health Center (FHC) model | Obj 1: examine aspects of planning and implementation of partnership with New York University dental school. Obj 2: discussion of phased approach - business development, planning, implementation: key personnel, school selection, student selection, costs & cost-effectiveness. | Primary and secondary public school | Descriptive, retrospective |  |
| Mattheus (2024); US | Multiple schools in Hawaii, 6-50 schools (progressive growth over 3 years) | Healthy and Ready to Learn Program partnership with Hawaii Dental Service | School-based dental sealant program (SBDS) | Primary schools | Descriptive study Quantitative data: number of students served  Qualitative data: program evaluation | >50 surveys by school administrators, teachers and nurses who participated in the program |
| McCann (2020); US | Case study, county level, successful 5-year project |  | Describe successes, lessons learned | High (secondary) school | Description of implementation |  |
| Mohlabi (2010); South Africa | Region - 2 provinces, 5 districts in each; identify and describe the barriers that may hamper successful implementation of SHS at all levels of governance; recommend strategies to overcome such barriers. limited generalizability; | HPS | Comprehensive SHS: Obj 1: examine perceptions of barriers by various stakeholder groups. Obj 2: discuss current practices for areas of improvement. Three topics: • What are the barriers hampering the successful implementation of comprehensive school health services in your province? • How can these barriers be overcome? • How can the present systems of operation be improved to accommodate the implementation of comprehensive SHS programs in your province? |  | Qualitative, exploratory & descriptive, 10 focus groups - participants- 5 from 5 districts in Mpumalanga and Guateng provinces. 45-minute discussions, broad topics: identification of barriers of implementation, strategies to overcome identified barriers, improvement of current system to facilitate implementation. | n= 100 |
| Mojapelo (2019); South Africa | Ten schools, one school district, focus groups with school health teams | Integrated School Health Program (ISHP) | 1. What are the Southern District of Ekurhuleni's implementation constraints regarding the Integrated School Health Program (ISHP)? 2. What is the impact of identified implementation constraints or problems on the rendering of school health services in Ekurhuleni's Southern District? What ISHP implementation experiences have SHS had in Ekurhuleni? How can these constraints be alleviated in order to apply effective ISHP implementation and improvement strategies to benefit school children? | Primary & secondary | Descriptive study: Qualitative: focus groups with school nurses | N=24  School nurses and nursing assistants 3 groups (9,8,7) |
| Molete (2020); South Africa | Region - 10 schools in one province. limited generalizability; | HPS | Oral health program: Obj 1: assess implementation process against established policy recommendations Obj 2: identify current barriers and facilitators to implementation | Primary and secondary schools | Qualitative, case study. Observation of clinic environment, approximately 4 hours; Semi-structured participant interviews, 30-45 minutes, policy reviews. Data collection using process maps and interview guides | n=10 Dental hygienists |
| Nye (2011); US | One school – Clark Middle School | School-based health center focusing on sports physicals, immunizations, treatment of minor injury, medical referrals. Conceptual model: relationship between health status and academic achievement | Evaluation of pilot project. Focus on fidelity with implementation plan, identify strengths and challenges | Middle (secondary) school | Mixed methods  Quantitative = student, parent and staff surveys, document review  Qualitative = key informant interviews | 17 key informant interviews including medical providers, school staff, parents, stakeholders |
| Palme (2023); US | Primary and secondary schools in underfunded urban locations, interviews conducted over 3 mos. In 2021 | School-Based Health Centers (SBHCs) | Logistics of establishing SBHCs | Primary and secondary schools | Qualitative: thematic analysis of interviews | 9 hospital & 6 school employees |
| Pearrow (2016); US | School districts in city of Boston, Massachusetts. Development of school psychologist designed multi-tiered levels of support through school-based screening, interventions and community partnerships. Community needs assessment began in 2012 with implementation following pilot of 10 schools. | Comprehensive Behavioral Health Model. Integrates social, emotional and behavioral health services into schools by expanding the role of school psychologists and social workers. | Student mental and behavioral health. | Unspecified; public | Strategy document | Executive work group of Boston school psychologists from Boston Public schools, Boston Children's Hospital, and the University of Massachusetts Boston, school psychology program. Number unspecified. |
| Perreault (2013); US | Single small rural school district | School-based mental health program;  California Healthy start program | Research question: what constitutes a successful school based mental health program for all students | Elementary and junior high (primary and secondary schools) | Mixed methods Quantitative: Comparison of academic achievement in schools with SHMH program and those without.  Qualitative: key informant interviews and focus group | 14 key informant interviews including MH staff, district administrators, teachers |
| Rosen (2023); Tanzania | In depth interviews of 46 program implementers in Dar es Salamm (urban) and Njombe regions (rural) of Tanzania. Level of research: region - Dar es Salaam and Njombe regions. Voluntary program. Program occurred between November 2019 and March 2020. 24 participants from Dar es Salaam, 22 from Njombe. | HPV Plus. Adolescent health services. HPV Plus is a school-based integrated service delivery model. Includes health education for female students 10-14 years, nutritional assessment, visual acuity screening, deworming prophylaxis, and HPV vaccination for females 14-year-olds. | Immunization with HPV adolescent girls with coverage rate of 85% by 2025. Research aim was to understand barriers and facilitators to delivering HPV vaccination alongside routine adolescent health services to meet national immunization coverage targets. | Secondary; unspecified | Qualitative descriptive evaluation of enablers and barriers to delivering HPV Plus program | n=46 Program implementers and planning stakeholders. Vaccinators, healthcare workers, teachers |
| Saito (2015); Lao PDR | Country wide including national, provincial, district, school | Implementation framework: National School Health Policy based on recommendations of the Global School Health Initiative and WHO HPS | Implementation of National School Health Policy | Primary | Qualitative, descriptive evaluation of implementation of National School Health Policy. Stratified, purposive sampling. Key informant interviews and document review. Data analysis = content analysis using Whitman’s (2009) framework of successful policy implementation. | N=20  Program implementers at 4 levels (national, provincial, district, school) in 3 areas (north, central, south) |
| Seelam (2021); India | A realist evaluation to identify program aspects and their causal relationships with outcomes for eye health in reach program. six districts in India. exploratory study design and included data from multiple sources. | Refractive Errors Among Children (REACH) program | eye health, especially addressing refractory errors with spectacles. |  | A realist evaluation to identify program aspects and their causal relationships with outcomes for eye health in reach program. six districts in India. exploratory study design and included data from multiple sources. | six districts in India: Kanchipuram (Tamilnadu), Satara (Maharashtra), Satna (Madhya Pradesh), South 24 Parganas (West Bengal), Thrissur (Kerala), Virudhunagar (Tamilnadu); The program was expected to cater to more than 2 million children |
| Schäfer (2025); Europe | Secondary schools in high income countries | Research framework: Consolidated Framework for Implementation Research | Barriers and facilitators affecting implementation of school-based mental health interventions in secondary schools | Secondary schools | Systematic review of qualitative research, 26 studies included | 26 studies representing 5-8630 pupils |
| Shaibu (2010); Botswana | 27 schools located in the Gaborone district; 28 months of observation; interviews conducted but not recorded. | "School Health Policy". The SHP broadly comprises four elements: 1) detailed health assessments of pupils in their first and final years at primary school; 2) an immunization program; 3) a program of health education aimed at teachers and pupils; and 4) a detailed assessment of the school environment. | The purpose of the evaluation was to explore how SHS were delivered in schools in Botswana. The services listed in the manual include: dental care; environmental health; health education; immunizations; health surveillance; regular physical and mental health assessments. | Primary and junior secondary schools (7-15 years) | Data on current practices on SHS were collected using observations, field notes and interviews of various school personnel in 27 schools located in the Gaborone district, and surrounding villages using the assessment guide in the School Health Policy. Content analysis was used to analyze the data. | 27 schools |
| Shizume (2021); Japan | National focus with purpose to provide support for successful implementation of SHS in developing countries | Health Promoting Schools (HPS) and Focusing Resources on Effective School Health (FRESH) | Study aim: provide an overview of the Japanese nationwide SHS that has been in existence since 1872. Identify enabling factors that support successful implementation in developing countries. | Elementary and junior high schools (primary and secondary schools) | Narrative review approach to collect and review a wide range of sources including education related laws and notices, courses of study and manuals. Review of research papers and related documents | None |
| Shung-King (2013); South Africa | National; this is a comparison of two policies. | South African 2003 National School Health Policy and the revised 2012 Integrated School Health Policy (a health promotion and preventive school-based service); several key components: (i) a health assessment, whereby children are screened for a number of health conditions such as vision and hearing; (ii) health education and health promotion, age-appropriately tailored; (iii) psychosocial and mental health assessments; (iv) the identification and support of children with chronic health conditions; (v) facilitating the creation of safe and healthy school environments; (vi) preventive interventions, mainly immunizations and deworming (the provision of sexual and reproductive preventive services at school is still under contestation); and (vii) addressing minor ailments | This is a descriptive paper; the aim is to compare two policies and the degree to which they were implemented, but the methodology is unclear. | Ages 6-12 stated for deworming activities at one point. | Not stated. | None |
| Trivette (2003); US | County in central North Carolina. One alternative high school (for students who experienced incarceration, pregnancy, history of truancy etc.); two additional SBHCs (1x elementary and 1x middle school) opened later. | SBHC (comprehensive or total care health centers) | This is a descriptive paper. The aim is to describe implementation of a comprehensive SBHS | Elementary, middle and high school (primary and secondary schools) | descriptive paper; no respondents, no data collection | n/a |
| Watson-Jones (2016); Tanzania | Investigates which adolescent health interventions can be piggy-backed onto an existing HPV vaccination program for girls. Appears to reflect on national situation but no other information about number of schools etc. | Not stated | We explored the feasibility of integrating an adolescent health package with HPV vaccination in Tanzania through a 3 step process comprising a desk review, a national stakeholder meeting, and key informant interviews; we sought to identify interventions suitable to be added to HPV program; Interventions mentioned as being suitable for integration again included deworming; vitamin A supplements; health education, such as on menstrual hygiene, or condom demonstrations; supplements including iron tablets and folic acid; vision screening; TT vaccination; and HIV and family planning education. | Not stated, but HPV program targeted adolescent girls. | 3 project components; stakeholder meeting; 18 key informant interviews; a desk review of policies, guidelines and program reports. | 18 interviews with key informants |
| WestEd 2 (California Department of Education); (2019-2024); US | Paradise Unified School District | Not stated | School mental health systems | Elementary (primary) and High School (secondary) schools | Brief, statistics and descriptions of initiatives | 3 elementary (primary), 3 high school (secondary), 1 e-learning (unspecified), ~1600 students |

## **Table 3.** **Characteristics of included grey literature**

| **Authors** | **Country** | **Title** | **Year** | **Type of document (regulation standard, policy, framework, guideline, strategy, report, implementation, peer-reviewed article)** | **Geographic coverage (e.g. state, national, international)** | **URL or DOI** | **Document Summary** |
| --- | --- | --- | --- | --- | --- | --- | --- |
| Adeniyi (World Federation of Public Health Associations Oral Health Workgroup) | International | WFPHA Global School Oral Health Casebook | 2024 | Report | International | <https://www.wfpha.org/global-school-oral-health-casebook/> | Compilation of cases to showcase innovative school-based oral health programs worldwide. Recommendation for strengthening intersectoral collaboration, developing scalable models, expanding training to non-dental professionals, advocating for consistent funding and evaluation, and using policies as strategic tools for integration. |
| Government of Pakistan and United Nations Educational, Scientific and Cultural Organization (UNESCO) | Pakistan | School health program: A strategic approach for improving health and education in Pakistan. | 2010 | Strategy | Country | <https://healtheducationresources.unesco.org/sites/default/files/resources/SchoolHealthProgrammePakistan.pdf> | Overview of SHS implementation with findings that include enablers and barriers. |
| Guinosso, S., Whitaker, K. & Dyer, J. | United States | The role of school-based health centers in the Adverse Childhood Experiences (ACEs) Aware Initiative: Current practices and recommendations | 2022 | Report | State | <https://www.acesaware.org/wp-content/uploads/2022/01/ETR-ACEs-Aware-SBHC-Practice-Paper.pdf> | Through California’s ACEs Aware initiative, the Office of the California Surgeon General and the California Department of Health Care Services lay out a roadmap to address ACEs in California through systemic reforms designed to prevent and screen for ACEs and treat toxic stress. The ACEs Aware initiative offers MediCal providers training, screening tools, clinical protocols, and payment for screening children and adults for ACEs (www.acesaware.org). |
| Kennedy Forum | US | Sustainable School-Based Mental Health Services: A Comprehensive Approach | 2024 | Report | National | <https://www.thekennedyforum.org/app/uploads/2024/07/Sustainable_School-Based-Mental_Health_Services.pdf> | Report outlining obstacles for sustainable mental health services in schools with actionable solutions at the Local, State, and Federal Level. Barriers of funding complexities, workforce shortages and administrative burdens should be addressed by leveraging diverse funding sources to support comprehensive financing plans, partnerships between schools, communities and the private sector and targeted advocacy. |
| Ministry of Education, Science and Technology, Ministry of Health, Ministry of Agriculture and Food Security (UNESCO). | Malawi | School health and nutrition (SHN) guidelines: Together for healthy productive schools. | 2009 | Guideline | Country | <https://healtheducationresources.unesco.org/sites/default/files/resources/malawi-school-health-and-nutrition-guidelines.pdf> | The document clearly defines what SHN providers should do at all levels to improve health status, environment and life styles of school aged children. |
| Ministry of Health, & Ministry of Basic Education. | South Africa | Integrated school health policy. | n.d. -2020 | Policy | Country | <https://www.childrenandaids.org/sites/default/files/2020-01/South%20Africa%20Integrated%20School%20Health%20Policy.pdf> | Outlines the role of our respective departments in addressing the health needs of learners, with the aim of ensuring that a strong school health service operates according to clear standards across the country. |
| Ministry of Health, State of Eritrea. | State of Eritrea | Strategic plan for the implementation of reproductive, maternal, newborn, child and adolescent health (RMNCAH) and healthy ageing (HA) programs in Eritrea 2022–2026 | n.d. - 2022 | Strategy | Country | <https://www.afro.who.int/sites/default/files/2022-09/2022_RMNCAH_Eritrea.pdf> | The focus of this new strategic plan is on the implementation of high-impact evidence-based RMNCAH & HA interventions across the life course. Guiding document for implementation. Focus on adolescent reproductive and sexual health. |
| National School and Adolescent Health Programme in the Ministry of Health and Sanitation | Sierra Leone | SHS policy. | 2020 | Strategy | Country | <https://mbsse.gov.sl/wp-content/uploads/2022/08/SCHOOL-HEALTH-POLICY-2021.pdf> | Sierra Leone’s first comprehensive School Health Policy and Strategy, to guide the systematic provision and scale up of comprehensive Sierra Leone SHS as defined in these documents. |
| Schools for Health in Europe Network Foundation. | Denmark | A qualitative exploration of barriers and facilitators for the implementation of SHS promotion in Europe. SHE Monitoring Report, Schools for Health in Europe. | 2021 | Strategy | International - European and Central Asia consortium | <https://www.schoolsforhealth.org/sites/default/files/editor/mapping/report_on_facilitators_and_barriers_2021.pdf> | Qualitative report from 12 stakeholders and health workers in 5 countries in the Schools for Health in Europe Network Foundation (SHE). Topic was understanding barriers and facilitators of implementation of school health promotion activities. |
| Substance Abuse and Mental Health Services Administration. | United States | Utah's school behavioral health services implementation manual. | 2010 | Strategy | Country | <https://digitallibrary.utah.gov/aw-server/rest/product/purl/USL/f/27762610-403b-468b-b731-44909ab1c7ee> | Grant funded project that utilized lessons learned from six partnerships of mental/behavioral health services that were integrated with school-based service delivery to develop an implementation guide for school behavioral health services. |
| Taofeek | Nigeria | Challenges Facing School Health Services in Secondary Schools in Ilorin South, Kwara State | 2024 | Publication (non-peer reviewed report of research study) | National | <https://www.researchgate.net/publication/387176342_Challenges_Facing_School_Health_Services_in_Secondary_Schools_in_Ilorin_South_Kwara_State> | Study reveals significant gaps in the availability and quality of SHS, including inadequate funding, shortages of trained health personnel, poor infrastructure and limited health awareness among students and staff. Addressing identified challenges requires a multistakeholder approach involving schools, government agencies, parents, and the community. |
| UNESCO, & FRESH M&E Coordinating Group | International | Monitoring and evaluation (M&E) guidance for SHS programs. Eight Core Indicators to Support FRESH (Focusing Resources on Effective School Health) | 2014 | Guideline | International | <https://healtheducationresources.unesco.org/sites/default/files/resources/FRESH_M%26E_CORE_INDICATORS.pdf> | Target audience: The FRESH M&E Guidance is targeted at governments and organizations in low- and middle-income countries intending to use this document to develop a national self-assessment tool to monitor and evaluate their SHS programs. It is also intended for program directors and managers to use this broad guidance to develop specific questionnaires for their assessments. Purpose: With its set of recommended indicators, the FRESH M&E Guidance intends to help programs ensure their implementation is more standardized and evidence-based and will allow easier comparative benchmarking and monitoring across countries. Additionally, it is hoped that the FRESH M&E Guidance will help lead to better coordination between programs and the priorities they address and ultimately contribute to better health and education outcomes. Use: The eight Core Indicators presented in this part of the FRESH M&E Guidance focus on national-level and school-level efforts to implement comprehensive school heath programs as defined in the international FRESH framework. |
| Washington School-Based Health Alliance | United States | Washington School-Based Health Alliance Toolkit | 2022 | Guideline | State | <https://wasbha.org/> | Guidance toolkit for implementation of SHS. Includes planning, collaboration, center design, community & stakeholder engagement, facilities, data & quality improvement, sample business plan. |
| World Health Organization. | Geneva | Global Accelerated Action for the Health of Adolescents (AA-HA!): Guidance to support country implementation. | 2017 | Strategy | International | <https://iris.who.int/bitstream/handle/10665/255415/9789241512343-eng.pdf?sequence=1> | Document on implementation guidance supports the implementation of the Global Strategy for adolescent health by providing the comprehensive information that countries need to decide what to do for adolescent health and how to do it. It builds on ongoing efforts to ensure that adolescents can Survive, Thrive and are in a position to Transform the societies in which they live. |
