# Supplementary material for: Enablers of and barriers to implementation of school health services: A scoping review
Source: PLoS One. 2026 Nov 13;21(11):e0361415. doi: 10.1371/journal.pone.0361415 (PMC13766220; doi:10.1371/journal.pone.0361415)
Supplement: Supplementary file 3 [file pone.0361415.s003.docx]

**Table 5.** Findings from the Literature: Enablers and barriers to implementing SHS

| **Preparing and Planning for SHS** | | | |
| --- | --- | --- | --- |
| **Enablers** | **Findings** | **Barriers** | **Findings** |
| Resource Allocation | Many enablers focus on securing funding or leveraging existing resources to support SHS initiatives. This includes utilizing federal, state, and local funding sources, as well as foundation funding and community partnerships. | Systemic Challenges | Many barriers stem from broader systemic issues, such as misaligned missions between health and education organizations, incompatible financing systems, and privacy concerns. There was also a lack of dissemination from national to local system levels. These systemic challenges can hinder collaboration and coordination efforts. |
| Collaboration and Engagement | Successful initiatives often involve collaboration between various stakeholders, including government agencies, healthcare providers, institutes of higher education, community organizations, parents, and educators. Engagement of these stakeholders and the community is crucial for planning, implementation, and sustainability. | Resource Constraints | Barriers include limitations in financial resources, staffing, and material resources, which can impede the planning, implementation, and sustainability of SHS initiatives. |
| Clear Purpose and Guidelines | Enablers emphasize the importance of having a clear purpose or goal for SHS initiatives, as well as guidelines or frameworks to guide implementation. This clarity helps in setting priorities, aligning efforts, and ensuring consistency. | Complexity and Compliance | Barriers arise from the complexity of implementing health programs within educational settings, including compliance with regulations, mandates, and standards. Short lead times, unfunded mandates, and lack of specific response plans contribute to these challenges. |
| Capacity Building and Training | Enablers include measures to build capacity among school staff, healthcare professionals, and community members involved in implementing SHS programs. This may involve training, education, and awareness-raising activities. | Community Resistance | Some barriers stem from social and cultural factors, such as parental or stakeholder objections to certain health services or misconceptions about their impact. Overcoming resistance and addressing misconceptions requires targeted communication and engagement strategies. |
| Innovation and Adaptation | Successful initiatives often involve innovation and adaptation to meet the unique needs and challenges of schools and communities. This may include leveraging new technologies, optimizing service delivery models, and adapting interventions based on local context. | Infrastructure and Logistics | Barriers include logistical challenges, such as transportation disruptions, supply chain issues, and limited infrastructure, which can affect the delivery of health services and the implementation of interventions. |
|  |  | Staffing and Training Challenges | Staffing challenges or inadequate coverage to meet students' health needs. Decreased staffing suggests a shortage of training and/or qualified healthcare worker professionals, potentially impacting the quality and accessibility of health services. |
| **Policy and institutional anchoring of SHS** | | | |
| **Enablers** | **Findings** | **Barriers** | **Findings** |
| National policy design | Enablers promoting institutional anchoring included explicit detailed policy element definitions with identified roles that contain specific responsibilities; and clear, distinct mission statements and health goals. Enablers also included funding mechanisms tied to specific program outcomes and frameworks that allowed flexibility without jeopardizing standardization. | Political support | Barriers most commonly noted were the lack of legislative requirements or mandates for services, inconsistent alignment of policy components across governmental sectors, and changing governing priorities over time. This political support also affects funding or financing elements that are essential for comprehensive implementation. |
| Stakeholder characteristics | Successful initiatives were supported by diverse groups of stakeholders from all formal and informal governance levels, across health and educational disciplines, and frontline implementers. This diversity promoted buy-in, partnerships, and meaningful collaboration. | Policy accessibility | Barriers result when national policies are inaccessible by implementers. Policy inaccessibility includes poor policy translation to practice, incomplete dissemination, or end-user unfamiliarity. |
| Common vision | Enablers underscore the critical aspect of a shared or common vision for program implementation. Understanding of the program's purpose across diverse disciplines and perspectives ensured a more complete implementation and support at levels beyond the individual school. | Locus of control | Conflicts arising from decision-making scope and accountability affects whether or not a policy is widely accepted across governing and stakeholder groups. Policy standardization that includes flexibility based on contextual factors are more likely to be broadly implemented. |
| Policy enforcement | Enablers tied to institutional anchoring identified mandated program implementation or evaluation criteria to monitor the implementation. This structure promoted partner accountability, standardized implementation across multiple venues, and maintenance of collaborations. | Siloed disciplines or sectors | The resulting barriers to policy and institutional anchoring are most often attributed to the lack of understanding between stakeholders operating in siloed yet parallel environments. Perspective-taking is critical to meeting shared vision and outcomes. |
| **Learning and management practices** | | | |
| **Enablers** | **Findings** | **Barriers** | **Findings** |
| Partnerships and Collaboration | This theme encompasses enablers such as partnerships for successful school-based vaccination programs, utilization of health centers for increased health-promoting activities, and involvement of non-profit governance boards and advisory groups. It highlights the importance of collaboration between various stakeholders to enhance the effectiveness of health initiatives in schools. | Resource Constraints and Workload Issues | This theme encompasses barriers such as funding constraints leading to access inequities, staffing shortages, and time constraints for completing tasks. It highlights the challenges associated with limited resources, both in terms of financial support and personnel, which can impede the effective delivery of health services in schools. Inability to complete the SHS program due to various barriers such as time limits, lack of staffing and lack of money. |
| Legislative Support and Policy Implementation | Enablers related to legislative support and policy implementation form another theme. This includes legislation documenting protocols for student concussion care, as well as the identification of a Minister for Health as a powerful champion. This theme underscores the significance of supportive policies and legislative frameworks in facilitating health interventions within educational settings. Clear legislation on the SHS that is being implemented facilitates understanding for all stakeholders, partners, and SHS health workers. | Data Management and Operational Challenges | The theme of data management and operational challenges includes barriers such as data sharing and interoperability issues, abstract nature of guidelines leading to differences in perspectives, and program delivery inconsistencies across schools. This theme underscores the difficulties in managing data, ensuring consistency in program implementation, and overcoming operational hurdles that hinder the smooth execution of SHS initiatives. School health providers’ lack of access to medical records from outside practitioners and outside practitioners lacking access to school health records. Programs that took over a year to implement or had challenges to implement lacked adherence and therefore did not continue. |
| Training and Capacity Building | The theme of training and capacity building encompasses enablers such as investment in training and ongoing coaching for stakeholders, health-care providers and SHS health workers, continuous coaching of district educational officers and teachers, and the establishment of a systematized supervision system. It emphasizes the importance of equipping personnel with the necessary knowledge and skills to deliver effective health services in schools. The ability to work with practitioners outside of the school setting and the school nurses or healthcare workers to ensure care coordination of SHS. | Training and Education Gaps | Encompassing barriers such as insufficient training duration, lack of quality control measures, and inadequate training to address sensitive issues like sex and reproductive health, this theme highlights the importance of comprehensive training and education for stakeholders involved in  SHS. It underscores the need for ongoing professional development to enhance knowledge and skills in addressing diverse health-related challenges. A lack of knowledge on the school nurses’ part on how to implement the program led to a barrier on the program’s sustainability. |
| Data Management and Monitoring | Enablers related to data management and monitoring form another theme. This includes the implementation of electronic data systems for improved data collection, as well as the utilization of sophisticated data management systems for tracking outcomes. This theme underscores the importance of robust data systems in monitoring the effectiveness and impact of SHS. | Stakeholder Engagement and Communication | The theme of stakeholder engagement and communication includes barriers such as lack of support by management, lack of understanding of services, and scheduling challenges for collaborative efforts. This theme emphasizes the importance of effective communication and collaboration among stakeholders, including school administrators, health professionals, and community members, to overcome barriers and ensure the successful implementation of SHS initiatives. |
| Holistic Approaches | Approaches such as the holistic view of learning/community school model, involvement of parents, students, and community members in advisory groups, and the importance of relationships across sectors at various levels were enablers for sustainability. Successful holistic approach also focused engaging the community and adopting holistic approaches that address not only health but also social and educational needs within school settings. | Coordination of School Events and SHS | Coordinating events considering scheduled school events suggests challenges in optimizing service availability and accessibility amidst other school activities. |
| **School physical environment** | | | |
| **Enablers** | **Findings** | **Barriers** | **Findings** |
| Physical Infrastructure Support | Having sufficient and appropriate space to provide the SHS facilitates healthcare delivery. Conducting physical needs assessments ensures that facilities meet the requirements for providing quality health services. Privacy-enhancing features such as partitions and noise cancellation machines create comfortable and confidential environments for health consultations. Safe storage for vaccines to remain at required temperatures. | Infrastructure and Resource Limitations | Lack of understanding and adherence to established (when available) SHS guidelines. Lack of toilet facilities, piped water, and environmentally unfriendly conditions point to inadequate infrastructure for maintaining basic hygiene standards. Maintenance issues, absence of policies, cost constraints, and lack of staff support collectively contribute to barriers in maintaining and sustaining SHS. Cold chain storage reliance on partners indicates a deficiency in infrastructure or resources necessary for proper vaccine storage. |
| Integration and accessibility | Having enough space for equipment and supplies. Locating health services on school grounds increases accessibility for students and staff. Efforts to make the health space welcoming promote student engagement and participation in healthcare activities. Telehealth initiatives facilitate communication and collaboration among stakeholders involved in student well-being. Group education sessions maximize efficiency in delivering health-related information and interventions. | Lack of appropriate school building structure | Lack of piping and toileting in the building or proper windows, as well as not having heating or air conditioning when appropriate were barriers. Lack of space to conduct the SHS or clinic which also included a lack of private space to provide the SHS. |
| Resource Management and Coordination | Quick access to essential medical supplies first aid kits or inhalers ensures prompt management of health issues. Involvement of program specialists assists in identifying needs, addressing challenges, and optimizing resource allocation. Clinic flow based on recommendations of health authorities streamlines operations and enhances service delivery effectiveness. | Confidentiality and Privacy Concerns | Factors making health spaces welcoming may inadvertently compromise confidentiality, posing challenges to privacy standards. Most buildings designed with open concepts, leaving few spaces for privacy, raise concerns about confidentiality and personal health matters. Lack of appropriate, private physical space for assessments compromises confidentiality and may deter students from seeking healthcare services. |
| Collaborative Partnerships | Involvement of both public and private primary and secondary schools fosters a comprehensive approach to SHS. | Service Availability and Accessibility Issues | No health appraisal, guidance, counseling, follow-up services, emergency care, or first aid availability indicates significant gaps in essential health services. Inadequate infrastructure, such as poor access to water, sanitation, and lack of wash basins, hinders the provision of basic health services. Lack of appropriate space limits the availability and accessibility of healthcare services within school premises. |
| **School social environment (relational and organizational context)** | | | |
| **Enablers** | **Findings** | **Barriers** | **Findings** |
| Collaboration and Partnership | Successful implementation of SHS requires positive social relationships among and between educational staff and healthcare providers and with the school community. Positive relationships are characterized by clear and accepted role definitions, effective communication, and shared goals. Sufficient workforce is an underpinning of successful relationships in the SHS environment. Involvement of various stakeholders such as school administrators, school counsellors, teachers, community resources, and health care professionals. | Resistance and Perceived Disruption | Resistance from various stakeholders, including teachers, school administration, and parents. It highlights how resistance to SHS, whether due to perceived disruption to the school day or lack of buy-in from influential figures, can impede efforts to promote health services within schools.  Differences between school and health systems, lack of clear and accepted role definitions and ineffective communication provide challenges. |
| Shared health goals | Health is seen as integral to the education of the students and there is professional satisfaction in working together to achieve positive outcomes for students and the community. Awareness and education of staff, students, and administrators on dating violence. Communication strategies to recruit teens to the service. Prioritization of student health within the education system, including curriculum focusing on lifelong activities. Attention to population to make health centers culturally safe for specific communities. | Lack of shared goals or expectations related to SHS | Barriers to implementing SHS occurred when school staff and/or stakeholders had different expectations of what SHS were or the activities that would take place. |
| Value and support of diversity and equity | A value in diversity is demonstrated by the recognition of cultural diversity in the community’s concepts of health and wellness and the ability to develop health services that are culturally acceptable to the community. There is the ability to adapt evidence-based programs to local needs. Utilization of local workers with local knowledge. Attention to cultural sensitivities, such as flying Aboriginal flags and choosing Aboriginal names for health centers. Collaboration between health professionals and families, addressing clashes in health models and beliefs. | Challenges of cultural diversity | There are challenges in developing SHS that are culturally acceptable. The healthcare workforce may not reflect the diversity of the school community population. This theme focuses on clashes between different cultural or belief systems regarding health and illness models. It underscores how cultural beliefs, including resistance from specific groups or faith-based schools, can pose significant barriers to the acceptance and effectiveness of SHS initiatives. |
| Persistence and Communication | Personal contact, persistence, negotiation, and undertaking in-depth consultations in advance. Ongoing positive working relationships among school nurses and physicians. Professional satisfaction in carrying out the activities of the SHS. | Circumstances where extremes of poverty and weak infrastructure exist; intersectional social factors that inhibit equitable access | Social conditions characterized by extremes of poverty and weak infrastructure found in low-income countries do not support the development of SHS. In high-income countries lack of equitable access created by intersectional social factors inhibit the fair distribution of SHS. |
| **Student participation** | | | |
| **Enablers** | **Findings** | **Barriers** | **Findings** |
| Students are actively involved in the development, implementation and evaluation of SHS | Students are purposefully engaged and empowered to be actively involved in the development, implementation and evaluation of SHS. Engaging students as health promoters increases accessibility and awareness of services. Surveying student attitudes and satisfaction allows for feedback-driven improvements. | Lack of purposeful engagement of students in planning, implementation and evaluation processes | Students are not purposefully engaged or empowered to be actively involved in the development, implementation and evaluation of SHS. Adolescents' exclusion from legislative and evaluative processes reduces their engagement and investment in health programs. |
| Services are easily accessed by students | SHS services are easily accessed by students with consideration of developmental, logistical and social factors. Students have direct access. SHS are private and confidential. SHS are paired with available infrastructure to enable participation. There is infrastructure for gaining consent. Direct access to nurses without referral streamlines healthcare services for students. Identification of eligible students for healthcare services improves accessibility. Service configurations that accommodate student schedules enhance availability. | Services are not easily accessed by students | SHS services have barriers to access that hinder students from participating. Consent and privacy concerns such as challenges related to obtaining consent, particularly from parents, may restrict access to services or delay care. Inadequate attention to confidentiality and privacy may undermine trust and discourage students from seeking assistance. |
| Empowerment and Engagement | Learning basic communication skills fosters empowerment among participants. Meaningful participation in planning empowers students and improves service effectiveness. Utilizing student champions and involving adolescents in strategic planning enhances engagement and ownership. Confidential and private systems promote a sense of empowerment and comfort among users. | Insufficient resource allocation | There are insufficient resources to accomplish meaningful student participation. Unfunded mandates pose financial barriers to implementation and sustainability. Staff workloads hinder efforts to involve students and adequately address their needs. Large class sizes limit individualized attention and support for students. |
| Tailored Support and Communication | Careful attention to consent and identification of specific needs ensures tailored support. Age/developmentally appropriate communication and educational materials facilitate effective communication. Personnel addressing behavioral/anxiety associated with dental care offer tailored support to students. | Social and Cultural Factors | Gender-specific restrictions on activities, such as recess programs, may create inequities and limit participation. Parental dominance in conversations with young children impedes student input and engagement. Fear of medical procedures and societal stigmas surrounding healthcare can deter students from seeking or utilizing services. |
| Community Collaboration and Awareness | Engaging school authorities, students, and local community field staff through common forums enhances awareness and acceptability of services. Utilizing user-friendly and confidential systems promotes community acceptance and utilization of services. Surveying student attitudes and satisfaction fosters community involvement and feedback. Collaborative efforts involving multiple stakeholders ensure service excellence and uphold standards. |  |  |
| **Involvement of parents and the wider community (partnerships and networking)** | | | |
| **Enablers** | **Findings** | **Barriers** | **Findings** |
| Parent Engagement with Community Engagement and Collaboration | Parents are broadly engaged in SHS implementation and health promotion, including working collaboratively with staff and the school, involvement in health and well-being committees, communicating directly with the leadership team and promoting healthy habits at home. Schools actively engage community stakeholders through effective communication and building good relationships. | Operational Challenges and Service Expansion | As school-based health programs expand, operational challenges emerge, affecting parent and community involvement. Longer wait times for appointments can arise as demand increases, potentially leading to dissatisfaction among service users. Resistance from some parents to following school policies may hinder the smooth implementation of health initiatives. Moreover, providing adequate translations for non-English speaking populations presents logistical challenges that can impede effective communication and engagement. |
| Accessible Support Services | No cost services were seen as an enabler: Schools are perceived as safe spaces offering accessible (free, onsite) advice and support for health and well-being to students and parents alike. In addition, support from external partners, such as governmental and non-governmental organizations, contribute to the implementation of health promotion strategies, enriching the support network available within schools. | Perception and Communication Deficits | Misconceptions and communication gaps pose significant barriers to parent and community involvement in school-based health programs. Parental perception that illnesses will be mild and vaccines ineffective may lead to underutilization of available healthcare services. Lack of comprehensive program introductions and specifics may result in misunderstandings and skepticism among stakeholders. Additionally, activities and awareness campaigns that fail to effectively reach communities limit their ability to engage with and benefit from the program. |
| Empowering School Ownership | Individual schools who maintain control and flexibility in the development and implementation of SHS initiatives, fostering a sense of ownership and autonomy proved successful. | Administrative and Structural Hurdles | Administrative and structural hurdles present obstacles to parent and community involvement in school-based health programs. The requirement for parental consent for enrollment and data collection can be a significant barrier, particularly when navigating complex consent processes. Limited community engagement and the spread of rumors may undermine trust in the program and deter participation. Furthermore, the absence of strategic advocacy and clear definition of essential services hampers efforts to address systemic issues and effectively allocate resources. |
| Strategic Communication and Outreach | Schools (including SHS health workers) who establish open channels of communication with parents, share SHS strategic plans publicly, and utilize media to raise community awareness found success. Having a key stakeholder/parent was useful in promoting engagement and awareness of SHS, as was using technology when available to assist parents in the knowledge and use of SHS. |  |  |
| **Sustainability** | | | |
| **Enablers** | **Findings** | **Barriers** | **Findings** |
| Resources | Critical resource factors include tangible items such as funding, human capital, and infrastructure, but also intangibles such as intellectual capacity, innovation, and time:  Adequate financial resources are available to support the implementation and maintenance of school health services (SHS). This includes budget allocations from governmental bodies, private donors, or grants.  Sufficient skilled personnel, including healthcare professionals, educators, and administrative staff, are available to deliver and manage the SHS effectively.  Schools have access to necessary physical infrastructure such as buildings, equipment, and technology to deliver health services.  There is a pool of knowledge and expertise among staff to develop innovative strategies, adapt evidence-based practices, and address emerging health issues.  Embracing innovation allows for the development of new approaches and technologies that enhance the effectiveness and efficiency of SHS.  Sustained commitment over time is ensured, allowing for the gradual integration and improvement of SHS within the school system. | Financial and Resource Constraints | Sustainability is impeded by financial barriers such as the high cost of additional support for school nurse positions, inadequate funding, insufficient training, reliance on short-term grants, and challenging billing practices, exacerbating issues like co-payments for medical clearance and constraints on reimbursement.  Financial barriers, such as inadequate funding and reliance on short-term grants, pose significant challenges to the sustainability of school health services. The high cost of additional support staff like school nurses, coupled with challenging billing practices and co-payments for medical clearance, exacerbates these constraints, limiting long-term sustainability. |
| Partnerships | Successful initiatives noted that effective, committed partnerships were instrumental in long-term sustainability. Participants included community members, educators, healthcare providers, policy makers, and organizational leadership:  Active involvement and support from the local community enhance the relevance and acceptance of SHS.  Collaboration with teachers and school staff ensures seamless integration of health services into the academic environment.  Partnerships with healthcare professionals and organizations extend the reach and expertise of SHS beyond the school setting.  Engaging policymakers at local, regional, and national levels ensures alignment with broader health and education objectives and secures necessary support and resources.  Strong leadership within schools and partnering organizations provides direction, advocacy, and accountability for sustaining SHS initiatives. | Regulatory and Workload Challenges | Regulatory burdens, including heavy caseloads hindering role expansion, increased workloads due to regulations, constrained public funding, and variability in fund utilization, pose significant barriers, alongside a lack of evidence-based outcome evaluation and policy misalignment.  Regulatory flexibility and alignment with the needs of school health services can facilitate sustainability. This involves reducing regulatory burdens such as heavy caseloads and allowing for role expansion of school health professionals. Additionally, evidence-based outcome evaluation can ensure efficient resource utilization and policy alignment, fostering sustainability. |
| Structure | Sustainable programs utilize theoretical frameworks, evidence-based guidelines, and a systematic approach during implementation and evaluation to retain program fidelity, promote accountability, and secure internal and external partnerships:  Adopting established frameworks and models guides the design, implementation, and evaluation of SHS, promoting consistency and effectiveness.  Using evidence-based practices ensures that interventions are grounded in research and have demonstrated effectiveness, increasing confidence and trust in SHS among stakeholders.  Implementing SHS systematically, with clear protocols, procedures, and monitoring mechanisms, enhances efficiency, quality, and accountability.  Maintaining fidelity to the intended model or intervention ensures consistency and effectiveness over time, even in the face of organizational or personnel changes. | Community Diversity and Service Coverage | Barriers to sustainability arise from community diversity, particularly language barriers, impeding effective communication and service provision, compounded by challenges in maintaining community ownership during expansion efforts and the absence of essential referral services, leading to limitations in service coverage due to financial, human resource, and logistic constraints. |
| Communication | Sharing of information throughout the planning and implementation process facilitates long-term sustainability despite contextual changes or differing perspectives. Examples of communication types include achievements, collaboration, financial and resource allocation, and progress and process monitoring:  Transparent and open communication among stakeholders fosters trust, collaboration, and collective ownership of SHS goals and outcomes.  Celebrating successes and milestones reinforces motivation and commitment to sustaining SHS efforts.  Facilitating collaboration among different stakeholders promotes synergy, innovation, and shared responsibility for achieving SHS objectives.  Transparent communication about resource allocation and budgeting ensures efficient use of resources and minimizes conflicts or misunderstandings.  Regular updates on progress, challenges, and lessons learned facilitate ongoing improvement and adaptation of SHS initiatives. |  |  |
| Shared vision | A common understanding of the purpose of SHS regardless of background, education, experience, position, or perspective is essential for long-term sustainability. Transparency, systematic approaches, joint planning, and diverse perspectives magnify the connection between the health and education of students:  Ensuring that all stakeholders share a common understanding of the purpose and goals of SHS fosters coherence, commitment, and alignment of efforts.  Openness and transparency in decision-making processes and priorities build trust and promote collaboration among stakeholders.  Using systematic planning and evaluation approaches ensures that diverse perspectives and priorities are integrated cohesively into SHS initiatives.  Valuing and incorporating diverse perspectives enrich the design, implementation, and evaluation of SHS, making it more responsive to the needs and preferences of diverse stakeholders. |  |  |
| **Intersectoral working between the two key ministries of MOH and MOE** | | | |
| **Enablers** | **Findings** | **Barriers** | **Findings** |
| Legislation | At the ministerial level legislation was a primary facilitator. This could be at a comprehensive school health services level, for example Japan’s school health service has been underpinned by a legislative framework for almost 150 years. Alternatively, legislation could be specific to identified health priorities. | Misaligned policy or legislation | Misalignment could occur as a result of assumptions, misunderstandings, and even incongruent regulatory requirements at regional and national levels. Misalignment could lead to legal action or liability concerns at the level of the school. |
| Collaboration and Partnership | Collaboration between Ministries of Health and Education, collaboration with local universities for nurse preparation, on-site collaboration between teachers and health professionals, stakeholder planning, and partnerships from the beginning, and partnership between education and health sectors. At the local level, strategic partnerships between health and education providers were a primary facilitator, but only where there was clear mutual benefit. A mutually beneficial strategic partnership was also evident where a hospital sought to reduce inappropriate presentations to their services. An inclusive leadership style was also crucial whether at the individual or lead agency level. Few articles specifically detailed how collaboration was developed. | Governmental and Bureaucratic Challenges | Limited or no government involvement in implementation. At the same time delayed implementation was often due to bureaucratic processes, poor collaboration between government departments, perception of SHS as a low priority at various administrative levels, and lack of communication leading to weak implementation. |
| Resource Allocation and Development | The addition of school nurses, formation of a task force, funding support from external sources, development of institutionalized human resources through training, and establishment of systems for sustainable human resource development for SHS between the two sectors was crucial. | Regulatory and Operational Constraints | Rigidity within heavily regulated education and health sectors was a common barrier. Other barriers included limited inter-sectoral convergence due to regulatory constraints, inequitable resource distribution, limited collaboration and understanding between Ministries of Health and Education, and limited familiarity with interventions outside respective areas of expertise. |
| Clarity of Purpose and Transparency | Positive perception of the role between health and education, clearly defined purpose, and measurable objectives, openness to quality improvement, and transparency in outcome results to the community, operation guidelines ensuring convergence and transparency, and personal relationships fostering collaboration. | Implementation and Coordination Challenges | Barriers to implementation included challenges in implementing coordinated SHS programs, limited inter-sectoral collaboration, inequitable resource distribution, poor collaboration between government departments at various levels, and limited collaboration and understanding between Ministries of Health and Education. |
